# A comparative structural analysis of the surface properties of asco-laccases

**DOI:** 10.1371/journal.pone.0206589

**Published:** 2018-11-05

**Authors:** Heidi A. Ernst, Lise J. Jørgensen, Christian Bukh, Klaus Piontek, Dietmar A. Plattner, Lars H. Østergaard, Sine Larsen, Morten J. Bjerrum

**Affiliations:** 1 Department of Chemistry, University of Copenhagen, Copenhagen, Denmark; 2 Department of Plant and Environmental Sciences, University of Copenhagen, Frederiksberg, Denmark; 3 Institute of Organic Chemistry and Biochemistry, University of Freiburg, Freiburg im Breisgau, Germany; 4 Novozymes A/S, Department of Agile Protein Screening, Bagsværd, Denmark; Universidade Nova de Lisboa Instituto de Tecnologia Quimica e Biologica, PORTUGAL

## Abstract

Laccases of different biological origins have been widely investigated and these studies have elucidated fundamentals of the generic catalytic mechanism. However, other features such as surface properties and residues located away from the catalytic centres may also have impact on enzyme function. Here we present the crystal structure of laccase from *Myceliophthora thermophila* (*Mt*L) to a resolution of 1.62 Å together with a thorough structural comparison with other members of the CAZy family AA1_3 that comprises fungal laccases from ascomycetes. The recombinant protein produced in *A*. *oryzae* has a molecular mass of 75 kDa, a pI of 4.2 and carries 13.5 kDa N-linked glycans. In the crystal, *Mt*L forms a dimer with the phenolic substrate binding pocket blocked, suggesting that the active form of the enzyme is monomeric. Overall, the *Mt*L structure conforms with the canonical fold of fungal laccases as well as the features specific for the asco-laccases. However, the structural comparisons also reveal significant variations within this taxonomic subgroup. Notable differences in the T1-Cu active site topology and polar motifs imply molecular evolution to serve different functional roles. Very few surface residues are conserved and it is noticeable that they encompass residues that interact with the N-glycans and/or are located at domain interfaces. The N-glycosylation sites are surprisingly conserved among asco-laccases and in most cases the glycan displays extensive interactions with the protein. In particular, the glycans at Asn88 and Asn210 appear to have evolved as an integral part of the asco-laccase structure. An uneven distribution of the carbohydrates around the enzyme give unique properties to a distinct part of the surface of the asco-laccases which may have implication for laccase function–in particular towards large substrates.

## 1. Introduction

Laccases (E.C. 1.10.3.2) possess a great biotechnological potential due to their ability to oxidize a wide range of phenolic substrates, spanning from small toxic phenols to large biopolymers such as lignocellulose. Like other multicopper oxidases, laccases operate via catalytic Cu-centers and coupling of the oxidative reaction to reductive cleavage of atmospheric oxygen into water. Laccases are produced by fungi, plants and bacteria and among their *in vivo* functions are lignin degradation, lignin biosynthesis, morphogenesis, pigmentation and pathogenesis [1, 2].

Laccases have been classified as members of the AA1 enzyme family of auxiliary activities in the CAZY classification system (Carbohydrate Active Enzymes Database; www.cazy.org; [3]). In this classification, enzymes are grouped in families based on similarities in sequence, and hence structure. Often further subgroupings correlate with differences in biological functions and substrate specificity. The two major classes of fungal laccases belong to AA1 subfamilies 1 (basidiomycetes) and 3 (ascomycetes), respectively, while subfamily 2 comprises ferroxidases and other laccase-like multi-copper oxidases.

With more than 100 depositions of laccases in the protein databank (http://www.rcsb.org/) the structures of laccases have been thoroughly investigated, and were recently reviewed by Hakulinen & Rouvinen [4]. The overall fold of the three domain laccases consists of three cupredoxin-like domains with the catalytic copper sites located at domain 3 (C) and the trinuclear copper centre located at the interfaces between domain 1 (A) and 3 (C). The catalytic centres comprise three distinct types of copper ions classified by their spectroscopic properties, an electron shuttling Type 1 Cu (T1), and a trinuclear copper cluster (TNC) formed by a mononuclear Type 2 Cu and a binuclear Type 3 Cu site [5]. In the analyses of laccase structure and function the catalytic copper sites and their immediate surroundings have attracted most attention [6, 7], often due to a desire to increase the redox potential and thereby expand the available substrate space of laccases for industrial applications. Small phenolic substrates that get oxidized have been shown to bind in a pocket near the T1-Cu site [8]. The size and topology of the T1 pocket vary significantly between phylogenetic subclasses of laccases. The recent observation of a binding site for the non-phenolic substrate ABTS localized 26 Å from the T1 pocket in a bacterial laccase [9], adds to the complexity of laccase function.

Laccases have also been shown to act on larger substrates, *i*.*e*. lignin modification and degradation. In contrast to other lignin degrading enzymes like peroxidases [10–12] who use H_2_O_2_ as the final electron acceptor, laccases use O_2_ as the final electron acceptor, which would facilitate industrial applications. Lignin comprises a large component of lignocellulose biomass and partial enzymatic degradation would have great implications for lignin as a resource for fuels, chemicals and materials. Laccase-catalysed degradation of lignin is presumed to occur *via* mediators, and it is unknown whether a direct laccase-lignin interaction takes place during catalysis. In a recent review Munk *et al*. [13] addressed the question if laccases can catalyze bond cleavage in lignin, and their answer to the question is a no! However, there is clear evidence that laccases act on lignin by oxidizing the phenolic subunits of lignin to reactive intermediates, similar to the role played by laccase mediator systems (LMS) [14]. Studies involving different mediators have revealed significant variations in reaction pathways and the end products depending on the nature and properties of the mediator [15, 16]. It is evident that the enzymatic function of laccases has many more facets than simple “substrate-in product-out” catalysis. Proteins, including laccases, are prone to adsorp onto the surface of large insoluble biopolymers such as cellulose and lignin [17–19]. Such interactions can be either productive or non-productive, and the latter constitutes a major challenge in biorefinery processes *etc*. Notably, the adsorption of laccase onto lignin was shown to be strongly pH dependent [17], showing that surface charges are important for laccase-lignin interactions.

Another noteworthy aspect of the laccases that influences their surface properties is their heavy degree of N-glycosylation. The effect of glycosylation on the functionality of basi-laccases was investigated by Vite-Vallejo *et al*. [20] and Maestra-Reyna *et al*. [21]. These studies showed that enzymatically deglycosylated laccases had similar stability and activity on small substrates compared to the native enzymes. However, selective removal of the glycosylation sites by mutagenesis revealed that some of N-glycosylation sites were indispensable, and most mutants displayed somewhat compromised stability and/or activity. The glycosylation sites are generally not conserved between basi- and asco-laccases, and a possible role of glycosylation of the asco-laccases has not yet been investigated.

With the aim of elucidating the structure and function of asco-laccases, we have undertaken the structure determination of the asco-laccase from the thermophilic fungus *Myceliophthora thermophila* (*Mt*L) [22], The structure is known of four other asco-laccases (AA1_3): *Ma*L from *Melanocarpus albomyces* [23, 24], *Ta*L from *Thielavia arenaria* [25], *Ba*L from *Botrytis aclada* [26, 27] and *An*L from *Aspergillus niger* [28] (S1 Table). A significant evolutionary distance between the different asco-laccases is reflected in significant structural differences. However, all structures share the asco-characteristic features, compared to basi-laccases, such as an N-terminal extension and the C-terminal plug [24–26, 29]. All crystal structures of asco-laccases except *An*L display similar, but not identical, dimers that pack head-to-head with the T1-pockets from the two protomers facing each other. Whether this dimeric arrangement exists in solution and if it has any biological relevance remain an open issue [25, 26].

In the following we present a comparative structural and sequence analysis of the AA1_3 asco-laccases with focus on the surface properties, glycosylation, the role of four surface-exposed tyrosine residues, the substrate binding pocket and dimer formation.

## 2. Materials and methods

### 2.1. Protein production and purification

Recombinant *Mt*L was overexpressed in *Aspergillus oryzae* as described by Berka *et al*. [22] adjusted to industrial scale. Mutations were checked at DNA level, and some at amino acid level using tryptic digests followed by LC-MS/MS for identification. Fermentation broth was made available from Novozymes A/S. *Mt*L was purified from the crude extract by two rounds of size-exclusion chromatography (SEC). The concentrated crude extract was thawn from -80°C in a water bath, centrifuged for 10 min at 10 000 x *g* to remove any particles, and then loaded onto a 1000 ml Sephadex G-75 column. The resin was packed in a XK50/60 column and pre-equilibrated with SEC-buffer (50 mM NaH_2_PO_4_, 150 mM NaCl pH 7.0). Fractions displaying laccase activity were pooled, concentrated and subjected to a second round of SEC. The SEC fractions with laccase activity (see 2.6) were pooled, concentrated and buffer exchanged to 20 mM Tris-acetate pH 8.0. Aliquots were stored at -80°C. The concentration of purified *Mt*L samples was determined from A_280nm_ and the molar extinction coefficient for *Mt*L (ε_280_ = 45 500 M^-1^cm^-1^ [30]).

### 2.2. Gel electrophoresis

*Mt*L was characterized by denaturing SDS-PAGE run on a BioRad TGX 4–15% gradient gel and non-denaturing isoelectric focusing (IEF) using a NOVEX IEF gel pH 3–7, following the protocols given by the manufacturers. Protein bands were stained with Coomassie Brilliant Blue.

### 2.3. Analytical size-exclusion chromatography

The homogeneity of *Mt*L in solution was further probed by analytical size-exclusion chromatography on a Superdex 200 HR 10/30 column (Pharmacia). The column was equilibrated with 50 mM NaH_2_PO_4_, 150 mM NaCl, pH 7 and calibrated using a set of molecular weight protein standards (Bio-Rad) comprising bovine thyroglobulin (670 kDa), bovine gamma globulin (158 kDa), chicken ovalbumin (44 kDa) and horse myoglobulin (17 kDa). The *Mt*L sample and the standards were all eluted at 0.4 ml/min.

### 2.4. MALDI-TOF mass spectrometry

The mass of intact *Mt*L was measured by mass spectrometry using an Autoflex Speed MALDI-TOF Instrument (BRUKER) calibrated with Bovine Serum Albumin (BSA) as molecular weight standard (M^2+^ 33216 Da, M^+^ 66431 Da, 2M^+^ 132861 Da). *Mt*L was diluted in 0.1% tri-fluoro-acetic acid (TFA) to a final concentration of 20 μM, and mixed 1:1 with a saturated stock of α-cyano-4-hydroxycinnamic acid (α-CHCA) matrix in acetonitrile/TFA (33 v/v% / 0.1 v/v%).

### 2.5. Capillary electrophoresis (CE)

CE analysis was performed under native conditions with a HP^3D^CE instrument (Agilent Technologies). A 2.2 mg/ml solution of *Mt*L was injected and eluted using 20 mM Na/K-phosphate as running buffer. Experiments were performed at pH 5 and pH 8, respectively. The capillary (0.05 mm in diameter and activated with 1 M NaOH for 20 minuttes) was kept at 15°C, and a constant voltage of 25 kV was applied.

### 2.6. Activity assay

Laccase activity was measured using syringaldazine (4-hydroxy-3,5-dimethoxybenzaldehydazine; SGZ; SIGMA S7896) as substrate. The assay was performed at room temperature with 0.28 mM SGZ in 25 mM Tris-maleate buffer pH 7.0, in a total volume of 4.4 ml. For stabilization, a buffer supplemented with 50 g L^-1^ PEG 6000 was used for dilution of the enzyme [30]. A HP 8453 UV-VIS spectrophotometer from Hewlett Packard was used to measure the absorbance at 530 nm to follow the formation of the purple oxidation product (tetramethoxy azobismethylene quinone; TAQ; ε_530_ = 65 000 M^-1^ cm^-1^; [31]). The enzyme concentration was adjusted to obtain initial velocities (ΔA_530nm_ min^-1^) within the linear range (0.1 to 0.4 AU).

### 2.7. Crystallization

Initial crystallization conditions were identified from screening with commercial kits: JCSG+ (Qiagen), PACT (Qiagen), Crystal Screen (Hampton Research) and Index HT (Hampton Research) using an Oryx 8 robot (Douglas Instruments) and a protein concentration of 100 mg ml^-1^. The high solubility of the protein is likely due to the glycosylation of the protein. Crystals formed under several conditions, but most of them diffracted poorly. Crystals obtained from condition C2 (30% PEG400, 0.2M MgCl_2_, 0.2 M HEPES pH 7.5) in the JSCG^++2^ screen from Jena Bioscience were further optimized in a manual setup using grid screens (pH, salt and PEG 400), additives and variation of drop ratios. All crystallization experiments were performed at room temperature, and both sitting and hanging drop vapor-diffusion setups were tested. Replacement of MgCl_2_ with CaCl_2_ improved the quality of the crystals. The crystal used for data collection was grown in a hanging drop setup with 3 μl protein (66 mg ml^-1^) and 2 μl reservoir solution equilibrated over a 500 μl reservoir (0.1 M HEPES pH 7.5, 34% PEG 400, 0.22 M CaCl_2_, 0.05 M glycine). Attempts were made to soak the substrates 2,6-dimethoxy phenol (2,6-DMP) and 2,2’-azino-bis(3-ethylbenzothiazoline-6-sulfonic acid) (ABTS) into the crystals by adding solid substrate to the drop as described by Kallio *et al*. [8].

### 2.8. Data collection, processing, structure solution and refinement

Crystals were flash-frozen directly in liquid nitrogen. MASSIF-1 beamline [32] at the European Synchrotron Radiation Facility (ESRF), Grenoble, France was used for data collection on several crystals. Data were indexed and integrated using *XDS* [33] and *SCALA* [34] was used for data reduction (Table 1). Generally, programs from the CCP4 suite [35] were used for structure determination and refinement. The structure was solved by molecular replacement in *MOLREP* [36] using the *Melanocarpus albomyces* laccase (*Ma*L; 2Q9O, 75% seq. id.; [24]) as search model. Model building was performed in *COOT* [37] and both *REFMAC5* [38] and *PHENIX*.*REFINE* [39] were used for refinement. *PROCHECK* [40] was used for structure validation. The different datasets showed different degrees of radiation damage, as reflected in disorder at the TNC site and also reduction of disulfide bridges *etc*.

**Table 1:** Data collection and refinement statistics for the *Mt*L structure.

Protein Data Bank code	6F5K
Data collection	
Radiation source	ID30-A1 ESRF (MASSIF-1)
Wavelength (Å)	0.966
Temperature (K)	100
Detector	PILATUS3_2M
Space group	*C*222_1_
*a*, *b*, *c* (Å)	67.45, 128.43, 163.62
Resolution range (Å)	48.23–1.62 (1.68–1.62)^a^
Total No. of reflections	438378 (43342)
No. of unique reflections	85380 (8718)
Completeness (%)	99.5 (99.6)
Redundancy	4.9 (5.0)
〈 *I*/σ(*I*)〉	7.7 (1.6)
CC_1/2_	0.99 (0.68)
*R*_merge_	0.11 (0.87)
Overall *B* factor from Wilson plot (Å^2^)	19.7
Refinement	
Resolution range (Å)	48.23–1.62 (1.66–1.62)
No. of reflections, working set	85258 (6170)
No. of reflections, test set	4507 (310)
*R*_cryst_	0.150 (0.301)
*R*_free_	0.189 (0.315)
R.m.s. deviations	
Bonds (Å)	0.021
Angles (°)	2.085
Average *B* factors (Å^2^) / No.	25.4 / 5194—overall
Protein atoms	24.1 / 4376
Carbohydrate atoms	46.6 / 267 (21 moieties)
Metal ions	20.8 / 6 (4 Cu, 2 Ca)
Water molecules	33.6 / 535
Ramachandran plot	
Most favoured (%)	98
Allowed (%)	2

^a^ Values in the parentheses are for the highest resolution shell.

Refinement statistics and model data are summarized in Table 1 for the dataset that appeared to suffer least from the radiation damage. Anisotropic thermal displacement parameters were included in the refinement of the metal ions, *i*.*e*. the four copper ions plus two calcium ions from the crystallization condition. The *Mt*L structure is deposited in the Protein Data Bank (www.rcsb.org) with entry code 6F5K.

### 2.9. Graphics and comparative structural analyses

Structural alignments and graphics were made with PYMOL [41]. The structure-based multiple sequence alignment (MSA) was performed using Promals3D [42, 43]. Subsequently, minor adjustments of the MSA were made manually in accordance with the structural alignments made with PYMOL [41]. Jalview [44] was used to depict the MSA, including conserved residues, while further annotations were added in Powerpoint. Dimer interfaces were analysed using the “Protein Interfaces, Surfaces and Assemblies” (PISA) server http://www.ebi.ac.uk/pdbe/prot_int/pistart.html [45].

## 3. Results and discussion

### 3.1. *Mt*L production, characterization and crystallization

**Fig 1:**
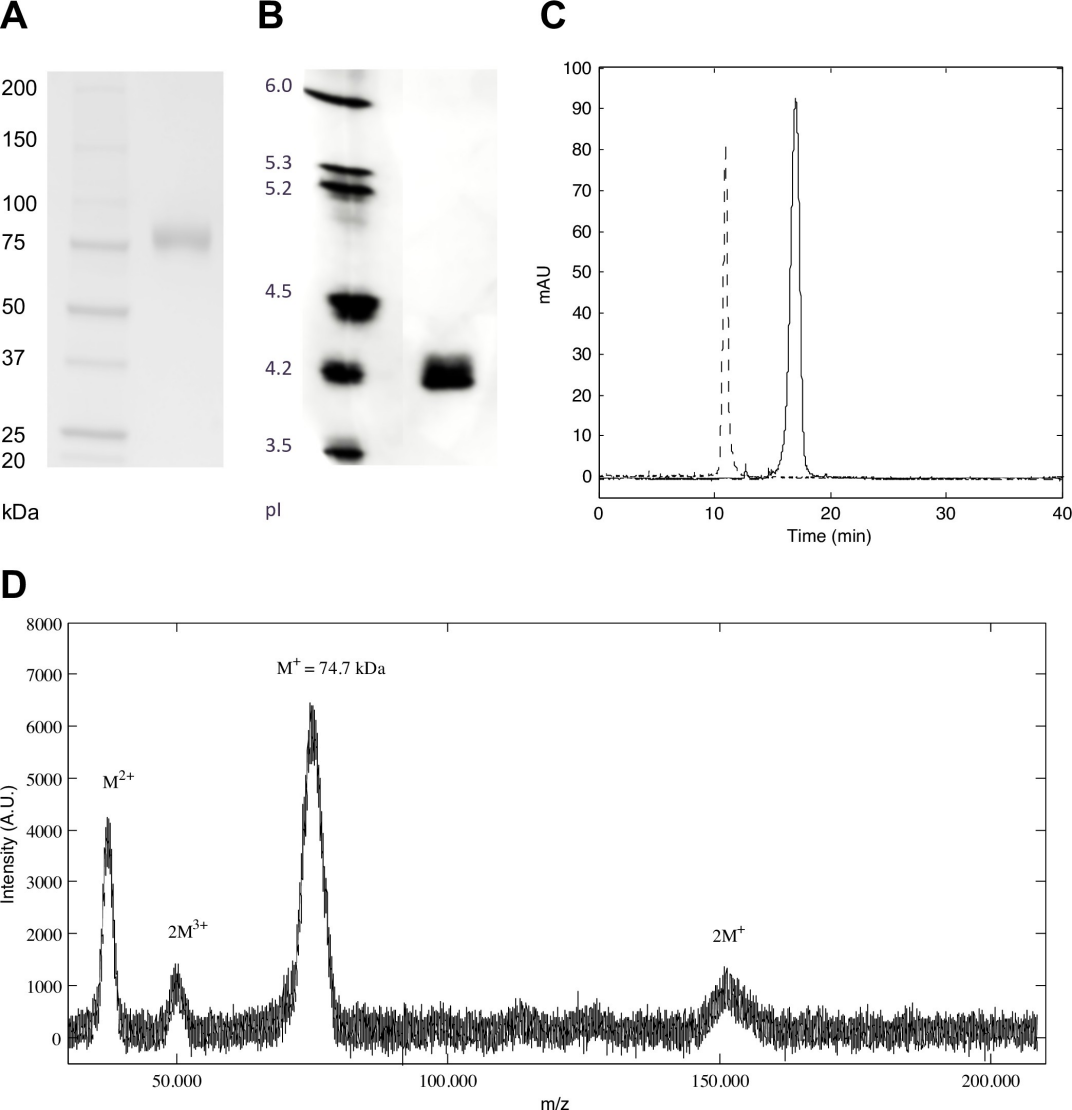
*Mt*L characterization. A) SDS-PAGE reveals a pure protein with an apparent molecular mass of 83 kDa. B) IEF analysis shows a highly homogeneous sample with a pI around 4.2. C) Capillary electrophoresis profiles of *Mt*L under native conditions, 2.2 mg/ml in 20 mM Na/K-phosphate buffers at pH 5 (*solid line*) and 8 (*dashed line*), respectively. In both cases a single peak is observed. D) MALDI-TOF spectrum of *Mt*L revealed that the mass of the intact protein is only around 74.7 kDa.

**Fig 2:**
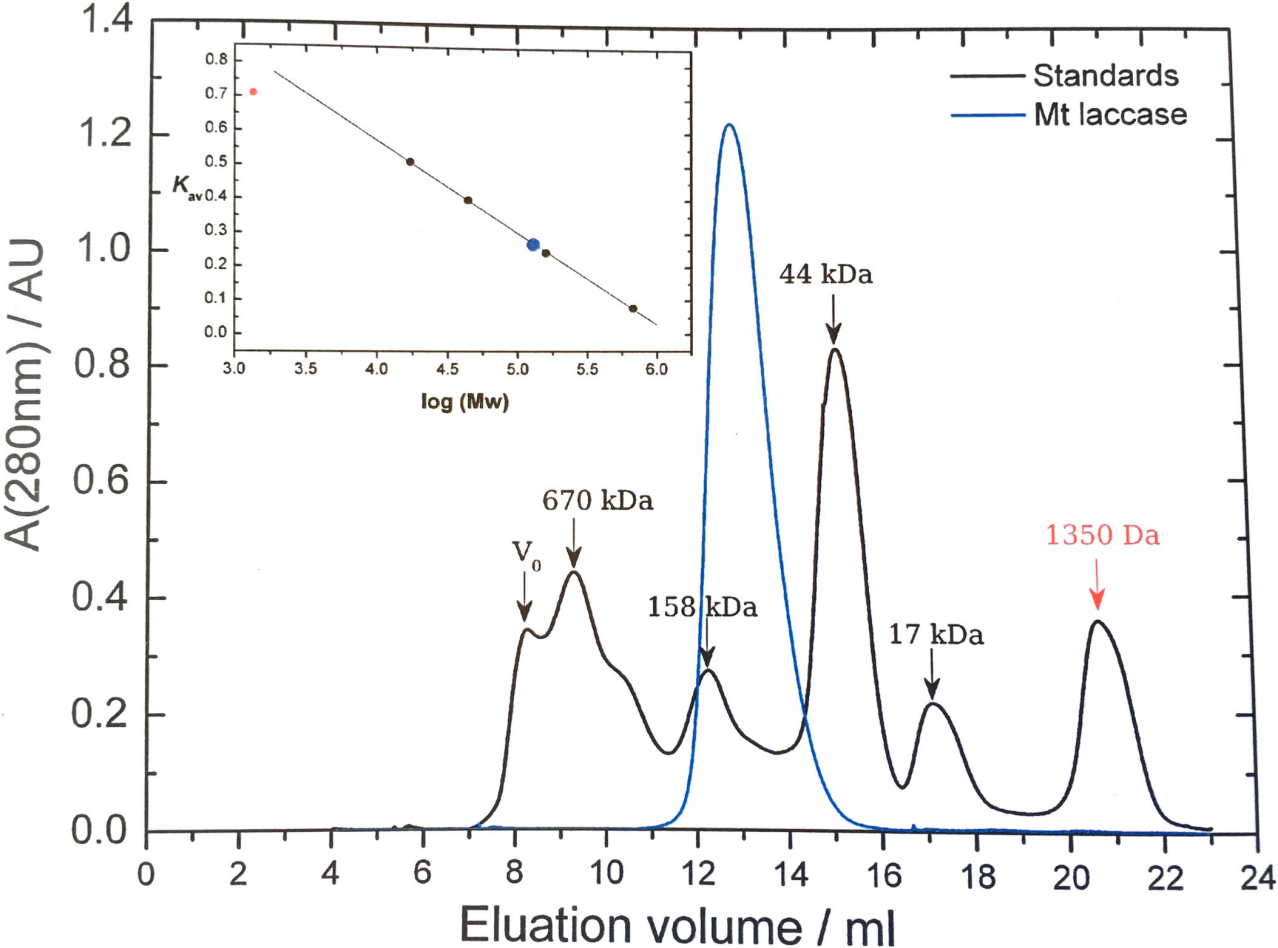
Size-exclusion chromatography analysis of *Mt*L. Analytical size-exclusion on a Superdex 200 HR 10/30 column equilibrated with 50 mM NaH_2_PO_4_, 150 mM NaCl, pH 7. Molecular weight protein standards: bovine thyroglobulin (670 kDa), bovine gamma globulin (158 kDa), chicken ovalbumin (44 kDa) and horse myoglobulin (17 kDa). The size estimate for *Mt*L is 128 kDa. The figure is adapted from [30].

Non-tagged *Mt*L was produced by heterologous expression in *A*. *oryzae*. Highly pure and active laccase was obtained from the crude extract by repeated size exclusion chromatography. The purified enzyme displayed a specific activity of 65 U/mg on the synthetic phenolic laccase substrate syringaldazine at pH 7, which is in good agreement with the activity data reported by Berka *et al*. [22]. Like the native enzyme, recombinant *Mt*L is a thermostable and heavily glycosylated protein, and it displays an activity half-life around 140 min at 65 ^o^C (S1 Fig). SDS-PAGE and IEF analyses revealed a highly homogeneous protein preparation with an apparent molecular mass of 83 kDa and a pI of 4.2 (Fig 1A and 1B). The homogeneity of the sample was further confirmed by capillary electrophoresis under native conditions and size exclusion chromatography that both showed a single peak (Figs 1C and 2), indicating that *Mt*L is present as a single species in solution. Based on the analytical SEC run, the size of *Mt*L in solution was estimated to be 128 kDa (Fig 2). This mass is larger than expected for a monomer, but too small for a dimer, suggesting that *Mt*L behaves differently than a typical globular protein in solution. Such deviations are not unusual and could be due to either a non-globular shape or ascribed to short-lived interactions between *Mt*L monomers in solution. As subsequently determined by MALDI-TOF mass spectrometry, the actual mass of the *Mt*L monomer is only around 74.7 kDa (Fig 1D), *i*.*e*. 8 kDa smaller than inferred from the SDS-PAGE analysis (Fig 1A). A similar discrepancy between the molecular mass estimated from SDS-PAGE and measured by MALDI-TOF, respectively, was also observed for a laccase from *Rigidoporus lignosus* [46], and it was explained as an unusual electrophoretic behaviour caused by the glycosylation. Given a theoretical mass of 61.2 kDa for the non-glycosylated protein, it could be inferred that *Mt*L is decorated with ca 13.5 kDa glycosylation. This high degree of glycosylation can explain why *Mt*L is highly soluble and could be concentrated to 120 mg ml^-1^ without any precipitation. Since purification and especially deglycosylation of laccases prior to crystallization often has been accompanied by loss of the labile T2-Cu [22, 26, 47–49], we aimed for crystallization of the fully glycosylated recombinant enzyme. A number of lead conditions were initially identified from sparse-matrix crystallization screening using commercial kits and a protein concentration of 100 mg ml^-1^. It is noteworthy that replacement of MgCl_2_ with CaCl_2_ led to well-diffracting crystals, which suggests specific metal ion interactions. Typically, clusters of large, but very thin, plates formed in 1–3 days. Glycine was identified as an additive which improved the morphology of the crystals, and further optimization led to the occasional growth of single crystals, often side-by-side with clusters of plates (S2 Fig).

### 3.2. Overall *Mt*L structure

**Fig 3:**
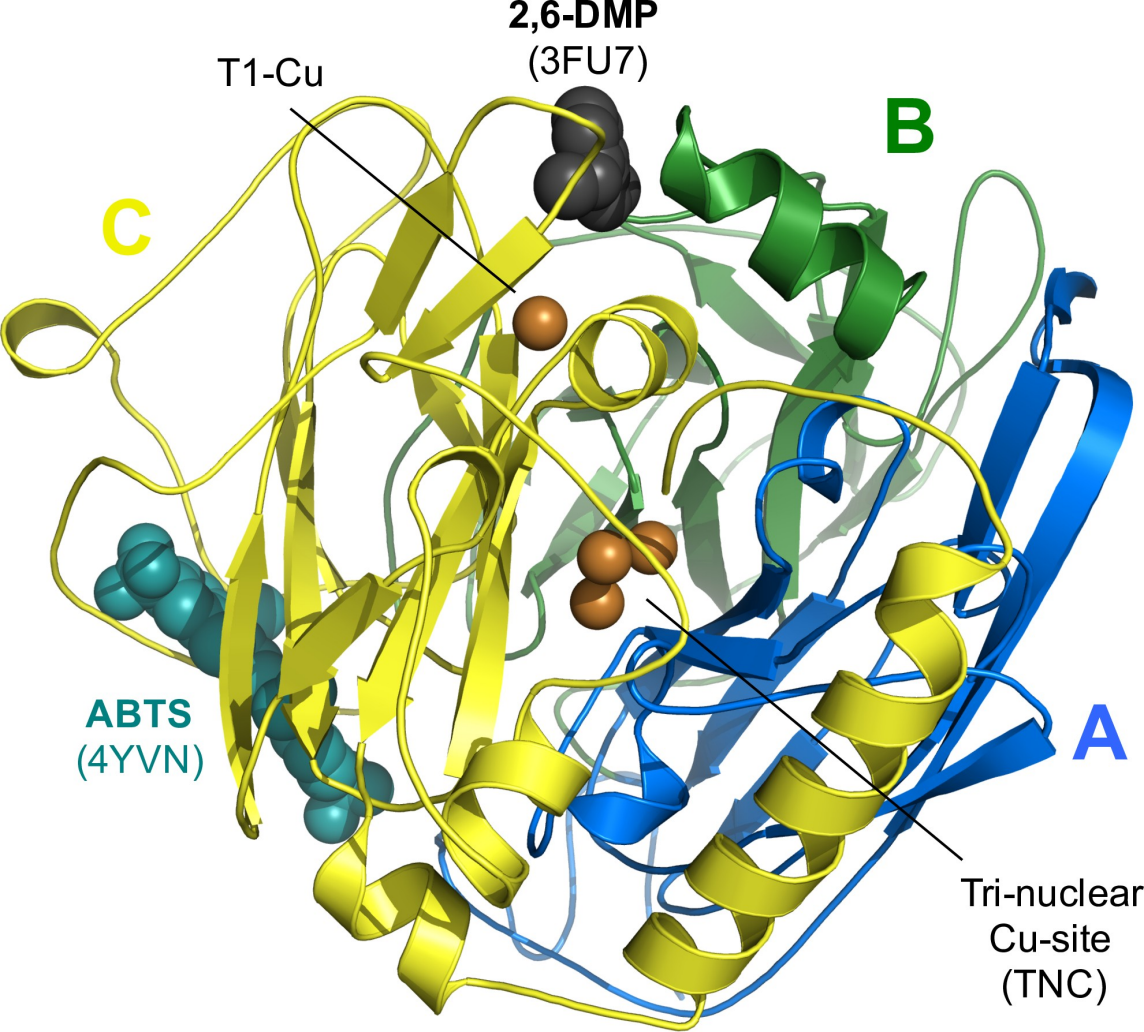
Overall *Mt*L architecture. *Mt*L structure with the three cupredoxin-like domains color-coded as follows: domain A (1–161) *blue*, domain B (162–341) *green* and domain (342–559) *yellow*. The four catalytic Cu ions are shown as *orange spheres*. Ligands from the PDB entries 3FU7 (*Ma*L:2,6-dimethoxyphenol; 2,6-DMP displayed as *black spheres*) and 4YVN (CotA:ABTS; ABTS displayed as *cyan spheres*) have been superimposed on the *Mt*L structure to illustrate the location of the T1-substrate binding pocket and an alternative substrate binding site, respectively.

**Fig 4:**
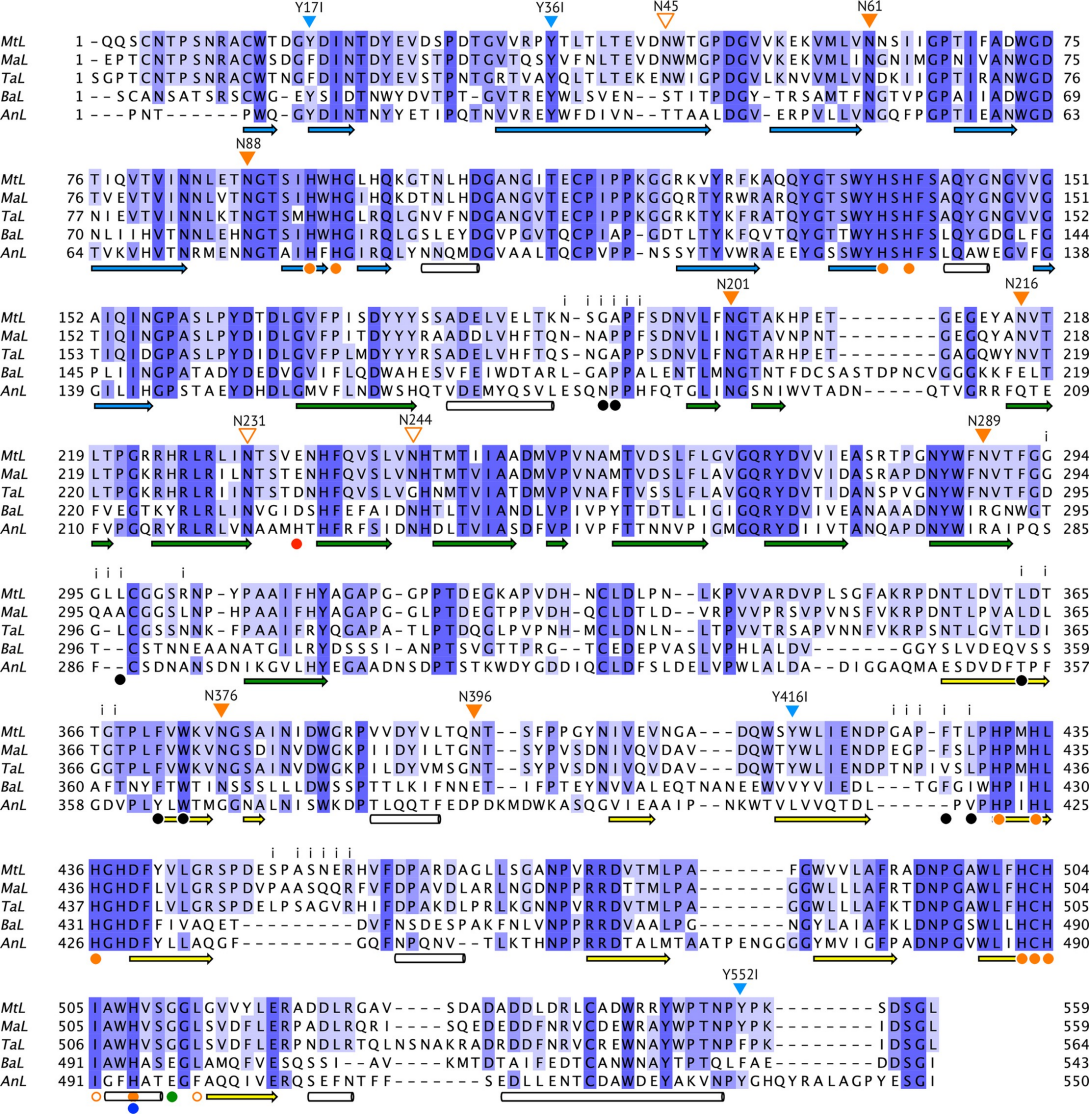
Structure-based alignment of *Mt*L, *Ma*L (2Q9O), *Ta*L (3PPS), *Ba*L (3QSR) and *An*L (5LM8). Structural features of particular interest in this study, such as glycosylation, dimer interface and *Mt*L variants, are indicated above the alignment. For *Mt*L, the putative consensus sites for N-glycosylation (N-x-T/S) are shown as *orange triangles*. The triangles are *filled* at the positions where glycan was observed in the structure. Residues located at the interface of the *Mt*L dimer are indicated with an “*i*”. *Mt*L variants discussed in the paper are denoted with *blue triangles*. Conserved secondary structure elements, domains, Cu-ligands and the residues forming the T1-pocket are shown below the alignment. β-Strands are color-coded corresponding to the three domains (A residues 1–161 *blue*, B residues 162–341 *green* and C residues 342–559 *yellow*) as in the Fig 3. Cu-ligands are represented with *orange circles*. *Open circles* indicate hydrophobic ligands like Leu513. Hydrophobic residues that line the T1-pocket, based on the *Ma*L:2,6-DMP complex [8], are indicated with *black dots* while His508 and Glu235, which form the polar recognition motif, are shown as *blue* and *red dots*, respectively. The extra polar residue, Glu497, in the T1-pocket of *Ba*L (and *An*L) is indicated with a *green dot*. Note that the N-terminal region of the *An*L structure is disordered and the residue numbers for *An*L in the MSA must be corrected (+27) to match the numbering in the mature enzyme sequence.

The crystal structure of *Mt*L was determined to a resolution of 1.62 Å by molecular replacement using *Ma*L (2Q9O; [24]) as search model (Fig 3). *Mt*L crystallizes in space group *C*222_1_ with one molecule per asymmetric unit. If glycosylation is not considered, the calculated solvent content is around 50%. However, given that each *Mt*L molecule is decorated with *ca* 13.5 kDa glycan, the actual solvent content must be significantly lower. In the crystal, N-linked glycosylation could be seen at seven out of ten consensus sites (N-x-T/S) (Fig 4). In total, 21 sugar moieties (3.9 kDa, ~ 29% of total glycan mass) were included in the model. The longest chain contains six sugar moieties. Additional difference electron density was interpreted as two Ca^2+^ ions originating from the crystallization conditions. One of these (CA605) is directly involved in crystal packing mediating contacts between two symmetry related molecules as it coordinates to Asp530 and Asp534 in one molecule and the carbonyl oxygen of Val341 in another molecule. The coordination sphere is completed by four ligating water molecules. CA606 coordinates to the carbonyl oxygen of Leu363 and five water molecules and may help to stabilize the local loop conformation. The interactions mediated by CA605 could explain the positive effect on the crystal quality observed for Ca^2+^ ions, due to their more variable coordination geometry [50].

Mature *Mt*L consists of 559 amino-acid residues [22]. Only the first residue Gln1 (not included in the model) and the side chain of Asp29 were not clearly defined. Considering the otherwise well-defined structure, relatively poor electron density indicated some mobility in the two loop regions Ser349-Arg354 and Pro461-Leu467. As expected, *Mt*L shares the overall architecture typical for 3-domain laccases [4]. It consists of three tightly associated cupredoxin-like domains (A, B and C; Fig 3), the secondary structural elements of the three domains are shown in Fig 4. The T1-Cu site is located in domain C, while residues from the A and C domains form the tri-nuclear copper (TNC) site.

### 3.3. Structural comparison of asco-laccases

**Fig 5:**
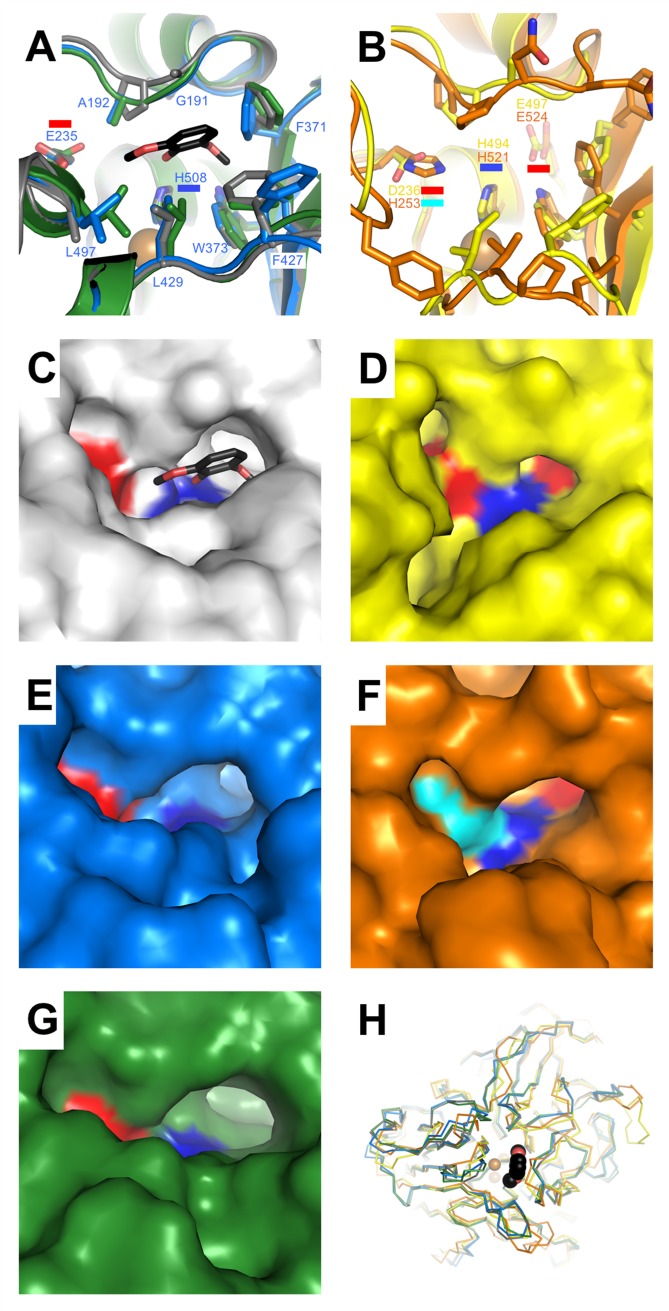
Phenolic substrate binding pockets in asco-type laccases. A) Superposition of *Mt*L (*blue*) and *Ta*L (3PPS, *green*) with the *Ma*L:2,6-DMP complex (3FU7, *grey*). *Mt*L residues lining the pocket are labelled. Glu235 and His508 that comprise the polar recognition motif are highlighted with *red* and *blue bars*, respectively. B) Superposition of *Ba*L (3SQR, *yellow*) and *An*L (5LM8, *orange*). The polar recognition motif and the extra polar residue, Glu497 (*Ba*L numbering), are indicated. C)—G) Surface representations illustrating the topology of the T1-pockets and mapping of polar residues. Color-coding as in A)—B). H) Structural superposition shown as *C*_*α*_*-traces* illustrating variations in loops around the T1-pocket. The ligand 2,6-DMP (*black spheres*) from 3FU7 indicates the location of the T1-pocket.

While the catalytic copper centres, including ten histidines and one cysteine that acts as Cu-ligands are strictly conserved, the comparison of the five asco-laccases with known structure (*Mt*L, *Ma*L, *Ta*L, *Ba*L and *An*L) reveals that this fungal subgroup is rather diverse, in terms of sequence, structure and function. The asco-laccases belong to the orders of Sordariomycetes (*Mt*L, *Ma*L and *Ta*L), Leotiomycetes (*Ba*L) and Eurotiomycetes (*An*L), respectively. At the sequence level, this is reflected in sequence identities ranging from 70–75% between laccases within the order of Sordariomycetes to 33–42% when comparing across the different orders (Fig 4 + S2 Table). Accordingly, *Mt*L superimposes very well with its closest homologues, *Ma*L with an r.m.s.d. of 0.60 Å between 521 pairs of C_α_-atoms and to *Ta*L with an r.m.s.d. of 0.78 Å between 522 pairs of C_α_-atoms (see Fig 5). In contrast, the more distant homologues *Ba*L, from the plant pathogen *B*. *aclada*, aligns with an r.m.s.d. of 0.73 Å for only 393 pairs of C_α_-atoms and *An*L, one of several laccases secreted by *A*. *niger*, aligns with an r.m.s.d. of 0.82 Å for only 359 pairs of C_α_-atoms (S3 Table). *Ba*L and *An*L share the same fold, however their loop regions show significant differences. The asco-laccase fold is stabilized by two conserved disulphide bridges (Cys114-Cys540 and Cys298-332, *Mt*L numbering). A third disulphide bridge (Cys4-Cys12) located in the N-terminal extension is conserved in all the structures, except for *An*L which has a disordered N-terminal extension that could not be modelled in the crystal structure [28]. *Ba*L displays a fourth disulphide bridge between Cys201-Cys209 that seems to stabilize an extended loop (see Fig 5). Among *Mt*L, *Ma*L and *Ta*L, the largest structural variations are observed for domain C. Most pronounced in the loop regions near the T1-substrate binding pocket (365–369, 424–429, 452–457) and the loops (410–414, 460–469, 347–351) near the putative, novel binding site described for ABTS [9] (see Fig 3).

### 3.4. Comparison of the T1-substrate binding pocket of asco-laccases

The shape of the pocket defined by several loops (vide supra) differs significantly between the basi- and asco-laccases, but also the latter group of enzymes shows significant differences. The T1-Cu is coordinated by two histidines (His431 and His508, *Mt*L numbering) and a cysteine (Cys503) in a trigonal planar arrangement; less than 4 Å from the Cu two non-polar residues are located in the axial positions. One of the axial ligands is invariably an Ile, while the nature of the other axial ligand varies. Oxidation of phenolic substrates occurs *via* electron transfer to the T1-Cu. The electrons are subsequently shuttled to the TNC-site where the reduction of molecular oxygen takes place [5]. Phenolic substrates are found to bind in a small pocket with the T1-Cu located at the bottom, as observed in the crystal structure of *Ma*L in complex with the substrate 2,6-DMP [8]. In this structure the phenolic substrate interacts directly with the T1-Cu ligand His508 (*Mt*L numbering), assumed to be the primary electron acceptor. Another residue that is highly conserved in the T1-pocket of fungal laccases is Glu235 (either Glu or Asp). This negatively charged residue has been shown to play an important role in the oxidation of phenolic substrates, possibly by facilitating proton abstraction [8]. Together His508 and Glu235, assisted by a bridging water molecule, form a polar site for phenol recognition. In most fungal laccase structures these are the only polar residues located in the otherwise very hydrophobic T1-pockets.

The redox potential of the T1-Cu has been correlated with the nature of the second axial non-polar ligand [51]. Typically, high redox potentials are reported for laccases from white rot fungi (basidiomycetes) (*ca*. 0.8 V; Phe in the axial position), medium potentials for asco-laccases (*ca*. 0.5 V; Leu in the axial position) and low potentials for laccases from plants and bacteria (< 0.5 V; Met in the axial position) [52, 53]. *Mt*L, *Ma*L *Ta*L, and *Ba*L have a Leu in the second axial position, *An*L resembles more the basi-laccases by having a Phe instead (Phe526). Based on the redox-potential-sequence-correlation described above, this could suggest that *An*L has a higher redox potential. However, the redox potential determined for *An*L is in the same range as for the other asco-laccases: *An*L 0.45 V [28]; *Mt*L 0.47 V [52]; *Ma*L 0.48 V [8] and *Ta*L 0.51 V [25]. On the other hand, a remarkably high redox potential of 0.72 V was reported for *Ba*L [26]. An observation that does not support the simple correlation between the redox potentials and the nature of the axial T1-Cu ligand.

The T1-pocket is largely conserved within the *Mt*L-*Ma*L-*Ta*L subdivision of asco-laccases (Figs 4 and 5A). However, the Leu297Ala substitution and differences in side chain conformations for Phe371 and Phe427 result in slightly different topologies of the substrate binding pockets (Fig 5C, 5E and 5G). The T1-pockets of *Ba*L and *An*L are distinctively different from the pockets of the other three laccases (Fig 5D and 5F). The variations in length and composition of the loops delineating the phenolic substrate binding site creates a unique topology for each of these enzymes. In *An*L Glu235 is replaced by a histidine (His253), and in both *Ba*L and *An*L an additional polar residue (Glu497 in *Ba*L) is found at the bottom of the pocket (Fig 5B, 5F and 5H); the other three asco-laccases contain a glycine in this position. Mutagenesis studies have shown that His253 in *An*L, similarly to Glu235, is essential for the oxidation of phenolic substrates [28]. The unique character of the T1-pockets in *Ba*L and *An*L demonstrates a previously ignored structural diversity within the asco-subgroup of laccases, that is reflected in the nature of the *in vivo* substrates. The preferred phenolic substrates for *An*L are 2-amino-phenols [28], which is perfectly matched with the presence of the carboxylic residue in the T1-pocket that can form hydrogen bonds to the amino-group. It confers also well with the observation that *An*L did not oxidize phenols with carboxylic acid substituents. This substrate discrimination is also facilitated by the overall negatively charged surface surrounding the T1-pocket in *An*L as suggested by Ferraroni *et al*. [28].

In the *Mt*L structure, the T1-pocket is devoid of any ligands, buffer components or cryo reagents. Instead it contains a network of water molecules. Despite the structural similarity between *Mt*L and *Ma*L, attempts to soak 2,6-DMP or ABTS into the *Mt*L crystals were unsuccessful. In the preparation of *Ma*L complexes with 2,6-DMP [8] small purple needle crystals formed in the drop after few minutes. Reproducing these experiments with *Mt*L crystals, gave small crystals of the product on the surface of the protein crystal, which itself did not gain any color, indicating that the reaction occurred on the surface rather than inside the protein crystal. Similar results were obtained from the attempts to soaks *Mt*L crystals with ABTS. Apparently, the *Mt*L crystal packing is incompatible with soaking of even small ligands into the T1-pocket. This could be due to the formation of unique dimers in the *Mt*L crystals.

### 3.5. Dimer formation of asco-laccases

**Fig 6:**
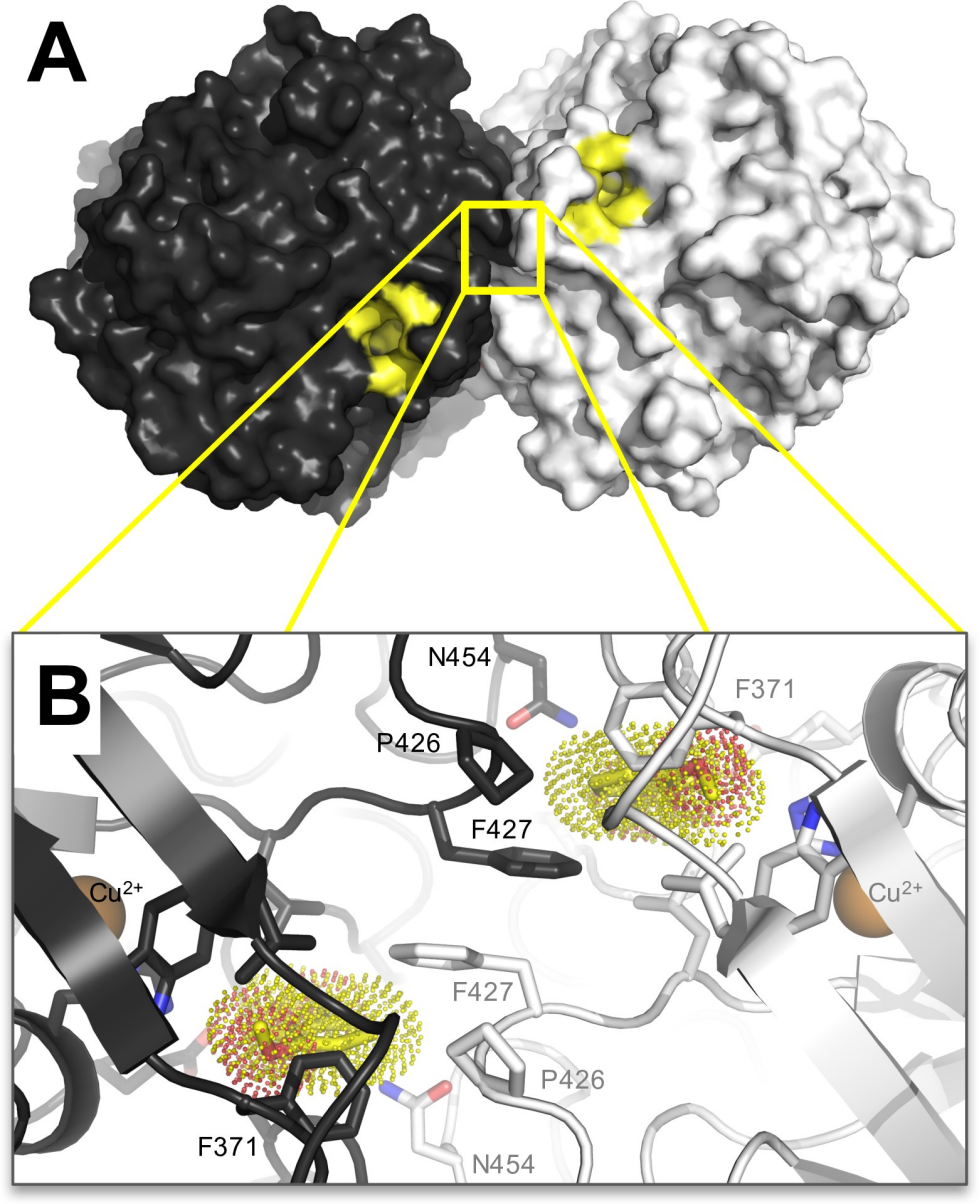
Dimeric *Mt*L assembly observed in the crystal. A) Surface representation with protomers shown in *black* and *white*, respectively. In this dimeric arrangement, the only entrance to the T1-pocket is through the conserved, (putative) substrate entrance channels [8] indicated in *yellow*. B) Zoom in on the dimer interface near the T1-pocket. Cartoon representation with selected residues shown as *sticks*. To mimick the position of a putative ligand, 2,6-DMP (*dotted surface*) from the *Ma*L complex (3FU7) was superimposed on each monomer separately. In the *Mt*L structure, the Phe427 docks into the T1-pocket of the other protomer and thereby hampering substrate binding.

All crystal structures of asco-laccases except for *An*L display similar, but not identical, dimers that pack head-to-head with the T1-pockets from the two protomers facing each other. This has led to speculations whether the dimeric arrangement exists in solution and if it has any biological relevance [25, 26]. *An*L also forms a dimeric assembly in the crystal, but this differs from the head-to-head arrangement described above and doesn’t directly involve the T1-pocket. The *Mt*L protomers are related by a twofold crystallographic symmetry axis parallel to *b*, show significant shape complementarity and a larger buried surface area than the *Ma*L and *Ta*L dimers, but similar to the *Ba*L dimer (Table 2). The residues forming the dimer interface in *Mt*L are indicated in Fig 4. The regions forming the dimer interface have low sequence conservation. It is interesting that the interface contains most of the loops that creates the T1-pocket of the *Mt*L structure, which is sealed by the dimer formation, allowing access only *via* a conserved narrow solvent channel (Fig 6A). It should be noticed that the conformation of Phe427 in *Mt*L differs from the one in *Ma*L as illustrated in Fig 5A. In *Mt*L it is docked into the substrate binding pocket of the other protomer (Fig 6B). Combined with a few other variations at the dimer interface, this creates steric restraints that seem to interfere with substrate binding. Based on these observations, we conclude that it is most likely that *Mt*L is active in its monomeric form. Neither did the analysis of the dimeric assemblies of other laccases, using the PISA server, support the existence of asco-laccase dimers in solution (Table 2), in contrast to the results from a SEC analysis of *Ba*L, which indicated the existence of a dimer [27]. The SEC and CE analyses of *Mt*L showed highly homogeneous preparations consistent with a single species in solution, although the existence of short-lived dimers cannot be excluded. Analytical size-exclusion chromatography suggested that *Mt*L is larger (128 kDa) than expected for a monomeric species (Fig 2). This size-estimate seems too small for an *Mt*L dimer but could be indicative for a fast monomer–dimer equilibrium in solution (minute scale) but could equally well be explained by a non-globular shape of the heavily glycosylated enzyme. Based on these data we conclude that *Mt*L is functional in its monomeric form, which also is the predominant form in solution.

**Table 2:** Dimer interface analysis (PISA server).

	Buried surface area (Å^2^)	Salt-bridges	Hydrogen bonds
*Mt*L	1000	2	6
*Ma*L	800	0	7
*Ta*L	673	0	6
*Ba*L	1007	0	2
*An*L	907	2	10

### 3.6. The TNC-site and disorder in the T2 water channel

The oxidation state of the copper ions in the TNC site and thus the nature of the ligand bridging the T3 and T3’ coppers are highly sensitive to the reducing properties of X-ray radiation [54, 55]. Consequently, a number of different states of the TNC-site have been observed in crystal structures of laccases, often with ambiguous electron density that reflects a mixture of different oxidation states and different reaction intermediates, as summarized in the recent review paper on laccase structures by Hakulinen & Rouvinen [4]. The effect of radiation damage was recently investigated in an interesting study of 17 structures of *Steccherinum murashkinskyi* laccase recorded with different degrees of X-ray exposure to obtain detailed information on the structural variations in the TNC site and the reaction mechanism [56]. The *Mt*L structure contains three fully occupied copper ions in the TNC-site. However, even the dataset that displayed the least radiation damage, revealed some disorder in this site. The site was modelled as a classical resting state with oxidized Cu ions, a hydroxide ion bridging the T3-coppers and a water/hydroxide ion coordinated to the T2-Cu (S3 Fig), as this appeared to be the major component present. This geometry is not in accordance with one of the intermediates described in Polyakov *et al*. [56] but rather a mixture of reaction intermediates.

The TNC-site is connected to the exterior through the T2-solvent channel [25]. In the *Mt*L structure, difference electron density suggests that several residues located in this channel, including His98, exist in multiple conformations. As the disorder appears complex and associated with a number of partially occupied water molecules, only the major conformers were included in the model. In the high-resolution structure of *Ma*L (2Q9O, 1.3 Å), His98 was found in its oxidized form, as an oxo-histidine, and in a conformation that blocked the T2-channel [24]. His98 is conserved in *Mt*L, where the electron density is also consistent with the presence of an oxo-His98, but in *Mt*L the residue is only partially oxidized and/or adopts multiple conformations.

### 3.7. The role of surface residues in the function of asco-laccases

**Fig 7:**
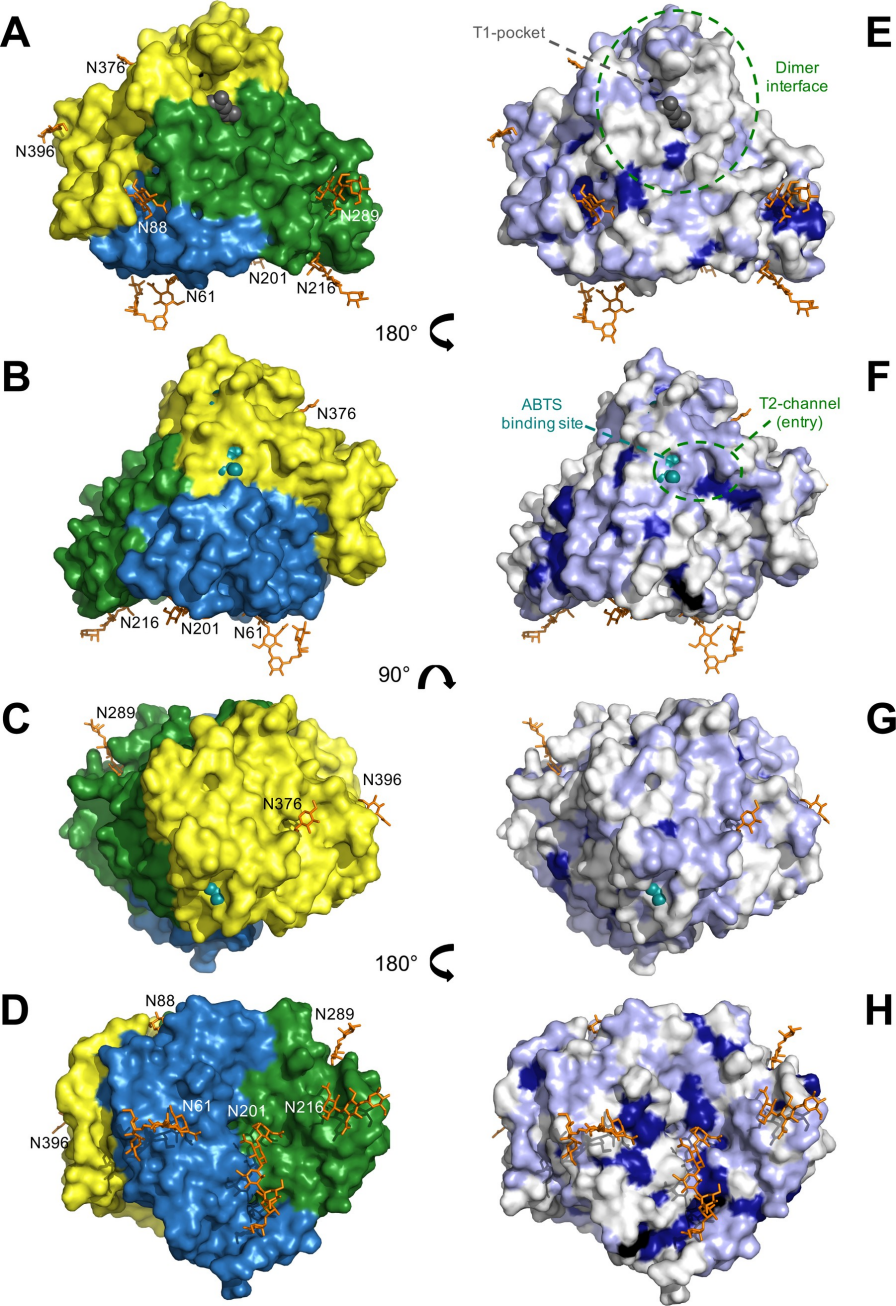
Conservation of glycosylation and surface residues between asco-laccases. A)- D) Surface representations of *Mt*L color-coded according to domains and showing the distribution of glycans around the protein. Glycosylation seen in the *Mt*L crystal structure are shown as *orange* sticks together with the numbering of the anchoring Asn residues. E)- H) Surface representations of *Mt*L with mapping of sequence conservation according to the MSA in Fig 4. Residues that are fully conserved among all five asco-laccases with known structures (*Mt*L, *Ma*L, *Ta*L, *Ba*L and *An*L) are shown in *dark blue* (including conservative substitutions Arg/Lys and Asp/Glu). Additional residues that are *only* conserved within the *Mt*L-*Ma*L-*Ta*L subgroup, but not in *Ba*L and/or *An*L, are mapped in *light blue*. Ligands from 3FU7 (2,6-DMP *black*) and 4YVN (ABTS *cyan*) are shown as space-filling molecules to indicate the location of the T1-substrate binding pocket and putative ABTS-site, respectively. Surface areas corresponding to the *Mt*L dimer interface and the entry/exit of the T2-solvent channel are indicated with *dashed green lines*.

For proteins, appearances matter. Thus, for laccases that act on large, complex lignocellulose substrates either *in vivo* or in biotechnological processes, not only the catalytic machinery involving the Cu-centres, but also the surface of the protein plays a role in enzyme function. Obviously, surface residues surrounding the T1-substrate binding pocket could affect substrate specificity, but also more distant sites may play important roles. Assuming that conserved residues play a role in biological function, we have investigated the sequence conservation among asco-laccases. The overall sequence conservation between the five asco-laccases with known structures is only 23% (Fig 4; 126 positions out of 559 residues). Most of these conserved residues are located around the catalytic Cu-sites and are buried inside the structure, and only few of the conserved residues are located on the surfaces. Mapping of sequence conservation onto the three-dimensional structure visualizes the conserved *vs*. variable surface regions (Fig 7). The four views of *Mt*L shown in Fig 7 differ significantly and show that the conserved surface residues are clearly not evenly distributed. The conserved residues are primarily found at the domain interfaces or comprise residues associated with the glycosylation of the enzyme (Fig 7E and 7H). It is also noteworthy that the “dimer interface” hardly displays any conservation (Fig 7E). This is consistent with our conclusion that the dimers only exist in the crystals, and the interface therefore is not a conserved structural feature. Notably, the surface that surrounds the T1-pocket is highly variable, even within the *Mt*L-*Ma*L-*Ta*L subdivision (Figs 4 and 7E). In fact, it is one of the regions with least conserved surface residues. Although variable in length and composition, the loops that create the T1-pocket are mostly comprised of neutral and/or hydrophobic amino acid residues, in all the asco-laccase structures except *An*L which has a negatively charged patch (Asp316, Asp380 and Glu493) close to the entrance to the T1-pocket that confers discrimination against phenolic acids as substrates due to electrostatic repulsion [28]. *An*L is in many ways a deviating asco-laccase and an example of how substrate selectivity may be achieved through evolution of surface residues surrounding the T1-pocket. An observation that could be exploited in rational enzyme engineering.

**Fig 8:**
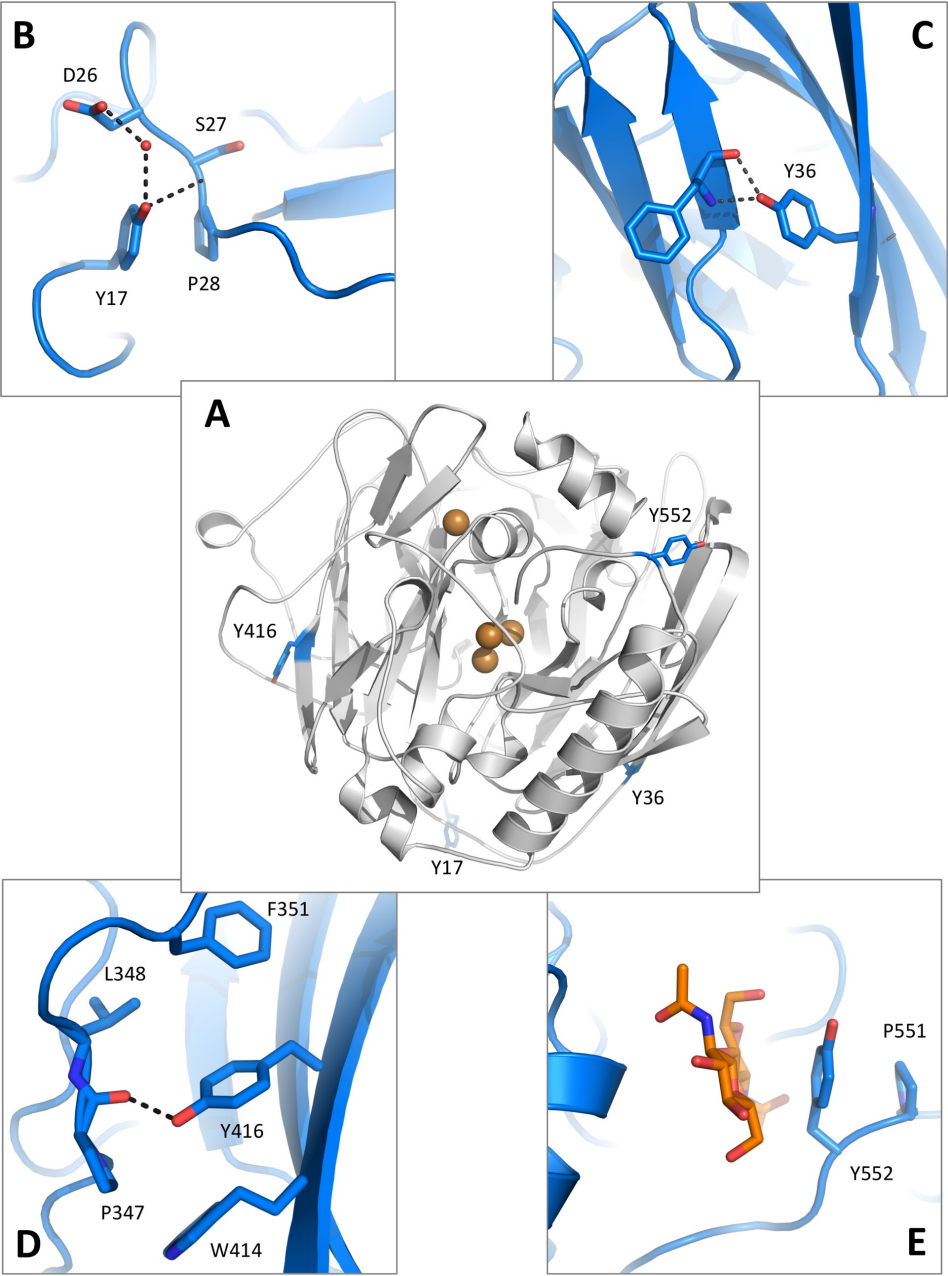
*Mt*L tyrosine variants. A) Location of the Tyr residues mutated to Ile in this study. The *Mt*L structure is shown in *cartoon* representation and Tyr residues as *blue sticks*. B) Tyr17 stabilizes loops in the N-terminal extension through hydrogen bonds and stacking interactions with Pro28. C) Tyr36 connects two β-sheets within domain A. D) Tyr416 packs in a hydrophobic environment and helps to stabilize a neighbouring loop *via* a hydrogen bond to the carbonyl oxygen of Pro347. E) The aromatic side chain of Tyr552 stacks between the glycan attached to Asn88 and Pro551 near the entry site of the C-terminal plug, shown to be essential for asco-laccase activity [29].

Tryptophans and tyrosines are reactive residues that may play roles in electron relay chains in oxidative enzymes [57, 58]. However surface-exposed tyrosines are also potentially susceptible to oxidation [59] and could form radicals resulting in polymerization, or be modified by oxidative processes as observed in the structure of the laccase from *Cerrena maxima* [60]. *Mt*L contains 11 tyrosine residues, four of these are located on the surface and either conserved or replaced by Phe in the other four asco-laccases, except for Tyr416 that is only conserved in the *Mt*L-*Ma*L-*Ta*L subdivision (Figs 4 and 8). To elucidate their role in the enzymatic function of *Mt*L, variants were prepared replacing Tyr with Ile. The variants were produced, purified and tested for activity using the SGZ-assay. The results are presented in Table 3, which shows that the replacement of the bulky hydrophobic Tyr to a less bulky, and non-aromatic, Ile results in a decrease in activity for all variants, with the Tyr17Ile variant as the least active. In addition to a possible role in charge transfer, an analysis of the *Mt*L structure showed that all surface-exposed tyrosine residues are engaged in intramolecular interactions as illustrated in Fig 8.

**Table 3:** Activity of *Mt*L variants in SGZ-assay.

Variant	U mg^-1^ (μmol min^-1^ mg^-1^)	Relative activity (%)
Wildtype	65.1	100
Y17I	0.01	0.02
Y36I	4.7	7.2
Y416I	6.1	9.4
Y552I	4.5	7.0

### 3.8. Effect of glycosylation of fungal laccases

Fungal laccases are secreted enzymes post-translationally modified with N-linked glycans. N-glycosylation can modulate the properties of a protein. In addition to increasing its solubility it may serve a number of important functions ranging from implication in folding, secretion, structural stabilization and enzymatic function [61]. Identification of glycosylation in a protein structure depends on the resolution of the diffraction data, interactions with the protein and the crystal packing. Therefore, the absence of well-defined electron density does not exclude the presence of glycans at a given site. Only a few systematic studies have addressed N-glycosylation of laccases [20, 21]. In these studies, enzymatically deglycosylated basi-laccase variants showed largely unchanged stability and activity on small substrates compared to the glycosylated laccase. However, selective removal of glycosylation sites by mutagenesis revealed that some sites were indispensable, and most of the mutants displayed somewhat compromised stability and/or activity. Our sequence and structural comparisons show that glycosylation sites are not conserved between laccases from the basidiomycetes and ascomycetes subgroups (S4 Fig). Since no biochemical information is currently available on the role of glycosylation of asco-laccases we have investigated this aspect of surface properties.

The amino acid sequence of *Mt*L contains 10 putative N-glycosylation sites with the consensus sequence Asn-X-Ser/Thr, more or less evenly distributed among the three domains (Fig 4). Based on MALDI-TOF MS data (Fig 1D) where the M^+^ ion had a molar mass of 74.7 kDa it is estimated that the recombinant MtL carries *ca*. 13.5 kDa of glycan, corresponding to 18% of the total molecular mass. In the crystal structure, 7 sites were found to be glycosylated (Table 4, Figs 4, 7 and S5 Fig). The attached carbohydrates are all consistent with a high mannose-type glycan (Asn-NAG-NAG-BMA-MAN_n_). At some sites, only the innermost N-acetylglucosamine (NAG) moiety could be modelled, whereas oligosaccharides containing five and six sugar rings, respectively, were visible at Asn61 and Asn201. The glycan linked to Asn61 is stabilized by crystal packing and only the innermost NAG-residue is involved in polar interactions with the protein. In contrast, all six sugar rings of the glycan attached to Asn201 interact with the protein *via* hydrogen bonds, hydrophobic interactions and/or water mediated contacts. This oligosaccharide packs against the protein in a long groove on the surface, at the interface between domain A and B (Fig 7D and 7H and S5C Fig). The NAG-NAG disaccharide linked to Asn88 is almost buried within the protein structure as it stacks between Asp181 and Tyr552 and displays a number of hydrogen bonds and water mediated contacts to protein residues. This sugar is involved in interactions with residues from all three domains and could stabilize the multi-domain structure (Fig 7A). Similarly, the two NAG moieties at Asn289 stacks between Trp287 and Tyr305 and form multiple polar interactions with Glu323 and the backbone carbonyl group of Tyr305 thereby stabilising two loops in domain B. The two NAG residues attached to Asn216 stack against His311 and form hydrogen bonds to His311 and two backbone amides in a glycine-rich loop in domain B (residues 313–318). At the Asn376 and Asn396 sites only one NAG residue was observed. These sugars are solvent-exposed and have few interactions with the protein. At the Asn376 site the N-acetyl group of NAG docks into a small hydrophobic pocket and forms a water-mediated contact to the carbonyl oxygen of Ile405, and the NAG moiety at Asn396 forms a short hydrogen bond to Tyr391. No glycans were observed at the putative N-glycosylation sites Asn45, Asn231 and Asn244. While Asn231 packs inside the protein structure, rendering glycosylation highly unlikely, the other sites could carry flexible glycans.

A sequence and structural comparison between *Mt*L, *Ma*L, *Ta*L, *Ba*L and *An*L revealed remarkable similarities in N-glycosylation of asco-laccases (Table 4). The sites at Asn88 and Asn201 are fully conserved, and the carbohydrates attached at these positions interact intimately with the protein as described above and also discussed for the *Ta*L and *Ba*L structures [25, 26]. Similar interactions are observed in all five structures, although variations are observed for the three outermost residues at the Asn201 site. The glycans at these two conserved positions are involved in stabilization of domains interfaces (A/B/C and A/B, respectively, Fig 7A and 7D) and appears as an integral part of the asco-laccase structure. The Asn376 site is conserved in four structures (all except *An*L). However, in this case the glycan displays only few interactions with the protein, and its role is unclear. The sites at positions Asn216, Asn289 and Asn396 are additionally conserved within the *Mt*L-*Ma*L-*Ta*L subdivision. In particular, the two NAG-NAG residues linked to Asn289 are highly integrated in the structure, they form almost identical interactions in all three laccases, and are thus likely to be involved in the stabilization of two loops in domain B. The glycan at Asn216 stabilizes a glycine-rich loop in *Mt*L and *Ma*L. In *Ta*L, the composition of the loop is different (Fig 4) and likewise the carbohydrate-protein interactions differ. The NAG-residue at Asn396 is solvent exposed and displays a few interactions with the protein, including a conserved hydrogen bond to Tyr391. However, this glycan is quite flexible, as reflected in the fact that it is only observed in one of the four *Ta*L monomers in the asymmetric unit of 3PPS, suggesting that it does not have a structural role.

Considering the relatively even distribution of N-glycosylation sites in the sequence, they display a remarkably uneven distribution at the surface (Figs 7A–7H), *e*.*g*. the part of the surface shown in Fig 7H differs significantly from the part in Fig 7G. Furthermore, the uneven spatial distribution of the glycans contributes to an overall non-globular shape, which could explain the overestimation of the molecular mass based on analytical SEC (Fig 2). Some surface regions are remarkably devoid of glycosylation and conserved residues, *eg*. the surface surrounding the T1-pocket and the surface around the entrance/exit of the T2-solvent channel (Fig 7E and 7F).

It should be mentioned that *Ba*L was reported to be heavily glycosylated and could only be crystallized after enzymatic deglycosylation [26]. Endoglycosidase H (Endo H) should trim the glycan down to the innermost NAG residue. Despite of this, longer glycan chains were preserved at the conserved Asn88 and Asn201 sites (*Mt*L numbering) in the crystal structure of deglycosylated *Ba*L (Table 3 and [26]), which illustrates that these glycans are protected by the extensive interactions with the protein. Deglycosylation of *Ba*L was accompanied by depletion of the T2-Cu, and a catalytically incompetent enzyme was obtained, as in other cases of laccase deglycosylation [47, 48]. It has led to considerations that the glycans exert a protective role, and it is their removal that causes loss of the T2-Cu. However, we do not find structural evidence that supports a Cu-protective role of the glycans. In asco-laccases no glycan is anchored near the T2-water channel that forms the access route to the T2-Cu site (Fig 7F). The same is the case for basi-laccases, as seen from a structural comparison including the crystal structures with PDB codes 2XYB, 3X1B, 1GYC, 2QT6, 3FPX, 3DIV, 1KYA, 2HRG, 4A2E, 2HZH and 1HFU. Depletion of the labile T2-Cu is more likely to be caused by the presence of the metal chelator ethylenediaminetetraacetic acid (EDTA) in commercial Endo H preparations, as has been argued by Ducros *et al*. [47].

**Table 4:** N-glycosylation observed in crystal structures of asco-laccases.

*Mt*L (6F5K)	*Ma*L (2Q9O)	*Ta*L (3PPS)	*Ba*L^#^ (3SQR)	*An*L (5LM8)
-	-	-	N39 (1)	N60 (2)
N61 (5)	-, *N39 (1)	-	N55 (1)	-, *N134 (1)
N88 (2)	N88 (3)	N89 (2)	N82 (3)	N103 (5)
N201 (6)	N201 (5)	N202 (6)	N194 (6)	N216 (3)
N216 (3)	N216 (2)	N217 (1)	-	-
+	N244 (1)	-, *N247 (1)	+	-
N289 (3)	N289 (3)	N290 (2)	-, *N305 (1)	-
N376 (1)	N376 (2)	N376 (1)	N370 (1)	-
-	-	-	-	^§^N400 (2)
N396 (1)	N396 (1)	N396 (1, mono B)	-, *N389 (1)	-

Number of carbohydrate moieties at each site is given in parenthesis. Legend: (-) the consensus site (Asn-X-Ser/Thr) is not conserved, (+) the consensus site is conserved, but no glycan seen in structure.

* Indicates the existence of an alternative site that is structurally close to the *Mt*L site.

^#^Note that these are glycans left after Endo H treatment [26].

^§^This site is not conserved in the other asco-laccases. Region with extended loops unique for *An*L.

## 4. Conclusion

The comparative structural analysis reveals that the asco-laccases comprise diverse group of enzymes. Despite the fact that laccases generally display broad substrate specificity and that 2,6-DMP is a typical laccase substrate (ortho-substituted phenol), the noted differences in the architecture of the T1-Cu pocket suggests some variability in the preferred substrates for the individual laccases. Our comparative study of asco-laccases shows an abundance of conserved N-glycosylation sites, which are spatially close and located in a surface area that is also rich in other conserved residues. This gives unique characteristics to this part of the surface which is likely to influence interactions with large biopolymers and may play a role in interactions with carbohydrate-rich substrates like lignocellulose. To understand laccase function in detail, it is therefore important that surface properties like glycosylation and electrostatics are taken into consideration.

## Supporting information

S1 Fig Thermostability of *Mt*L. The thermal stability of *Mt*L monitored as decay in activity after incubation at 65°C. Laccase activity was measured in 50 mM acetate buffer pH 4.5 using ABTS as substrate. The change in absorbance over time was measured at 415 nm to follow the formation of a blue-green product, thought to be the radical ABTS^+^ [62]. For stabilization, assay buffer supplemented with 50 g/L PEG 6000 was used for dilution of the enzyme [30]. The absorbance measurements were performed using a HP 8453 UV-VIS spectrophotometer from Hewlett Packard, and the enzyme concentration was adjusted to obtain initial velocities in the linear range (0.1 to 0.8 AU). The decay in activity at 65°C displays a half-life (t_1/2_) around 140 min.
(TIF)

S2 Fig Typical morphology of the *Mt*L crystals. The large single crystal was one of the crystals used for data collection (approximate dimensions 700 x 200 x 30 μm).
(TIF)

S3 Fig TNC-site in *Mt*L. Classical resting state geometry with oxidized Cu ions, OH- bridging the T3-coppers and water/hydroxide coordinated to T2. The Cu-Cu-distance is 4.6 Å.
(TIF)

S4 Fig **Mapping of N-glycosylation sites in selected asco- (A) and basi- (B) laccases.** Structure-based alignment where putative N-glycosylation sites with the consensus motif Asn-X-Ser/Thr are highlighted with *orange boxes*. For sites where attached glycans are observed in the crystal structure, the box is *filled*. Sites that are unlikely to be glycosylated, as they are either buried inside the protein structure or contain a Pro residue at the X1 site (Asn-Pro-Thr/Ser), are indicated with *boxes with a dotted line*. The alignment was made with Promals3D [42, 43] and is based on sequences and corresponding crystal structures of laccases from the basi-laccases *Pycnoporus cinnabarinus* (*Pc*L, 2XYB), *Rigidoporus microporus* (*Rm*L, 1V10), *Trametes sanguinea* (*Ts*L; no 3D structure), *Lentinus sp*. (Lcc4, 3X1B), *Trametes versicolor* (*Tv*Lx, 1GYC), *Lentinus tigrinus* (*Lt*L, 2QT6), *Trametes hirsuta* (*Th*L, 3FPX), *Cerrena maxima* (*Cm*L, 3DIV), *Trametes versicolor* (*Tv*L, 1KYA), *Trametes trogii* (*Tt*L, 2HRG), *Coriolopsis gallica* (*Cg*L, 4A2E), *Trametes ochracea* (*To*L, 2HZH), *Coprinus cinereus* (*Cc*L, 1HFU) and asco-laccases from *Myceliophthora thermophila* (*Mt*L, 6F5K), *Melanocarpus albomyces* (*Ma*L, 2Q9O), *Thielavia arenaria* (*Ta*L, 3PPS), *Botrytis aclada* (*Ba*L, 3SQR) and *Aspergillus niger* (*An*L, 5LM8). No glycans were modelled in the *Rm*L structure (1V10), but two sites were identified by MS-analysis [63]. No crystal structure is available for *Ts*L (UNIPROT C1JCL7), but this sequence is included as this is the laccase used in the (de)-glycosylation studies by Vite-Vallejo and coworkers [20].
(TIF)

S5 Fig Glycan-protein interactions in the *Mt*L structure. N-glycosylation sites A) Asn61, B) Asn88, C) Asn201, D) Asn216, E) Asn289, F) Asn376 and G) Asn396.
(TIF)

S1 Table Asco-laccases with known structure. (PDF)

S2 Table Pairwise sequence identity (%) between asco-laccases. (PDF)

S3 Table Structural alignment of *Mt*L with other asco-laccases. (PDF)
