# Supplementary figures and images for: A comparative structural analysis of the surface properties of asco-laccases
Source: PLoS One. 2018 Nov 5;13(11):e0206589. doi: 10.1371/journal.pone.0206589 (PMC6218047; doi:10.1371/journal.pone.0206589)

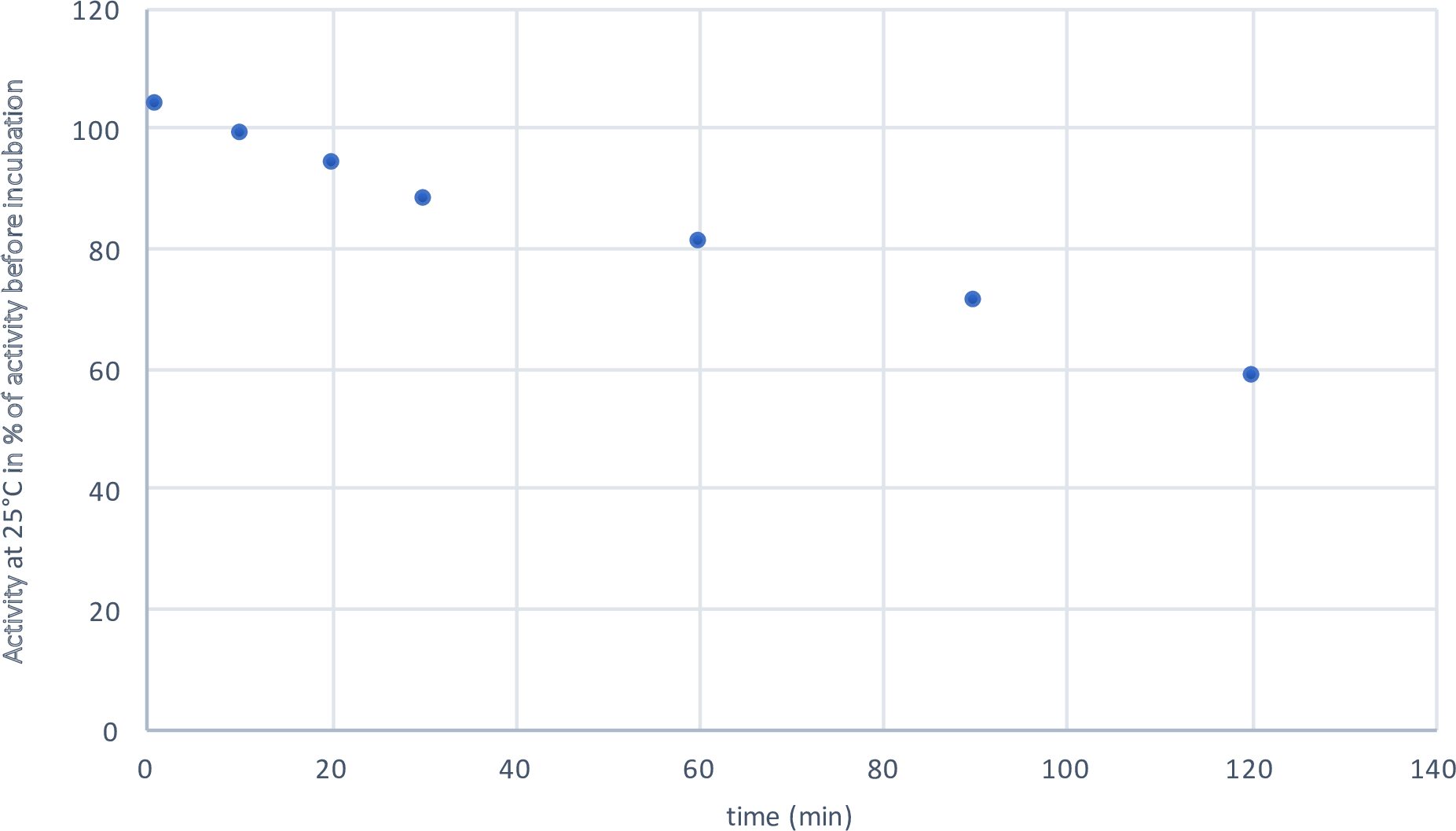

Supplement: S1 Fig — The thermal stability of MtL monitored as decay in activity after incubation at 65°C. Laccase activity was measured in 50 mM acetate buffer pH 4.5 using ABTS as substrate. The change in absorbance over time was measured at 415 nm to follow the formation of a blue-green product, thought to be the radical ABTS+ [62]. For stabilization, assay buffer supplemented with 50 g/L PEG 6000 was used for dilution of the enzyme [30]. The absorbance measurements were performed using a HP 8453 UV-VIS spectrophotometer from Hewlett Packard, and the enzyme concentration was adjusted to obtain initial velocities in the linear range (0.1 to 0.8 AU). The decay in activity at 65°C displays a half-life (t1/2) around 140 min. (TIF) [file pone.0206589.s001.tif]

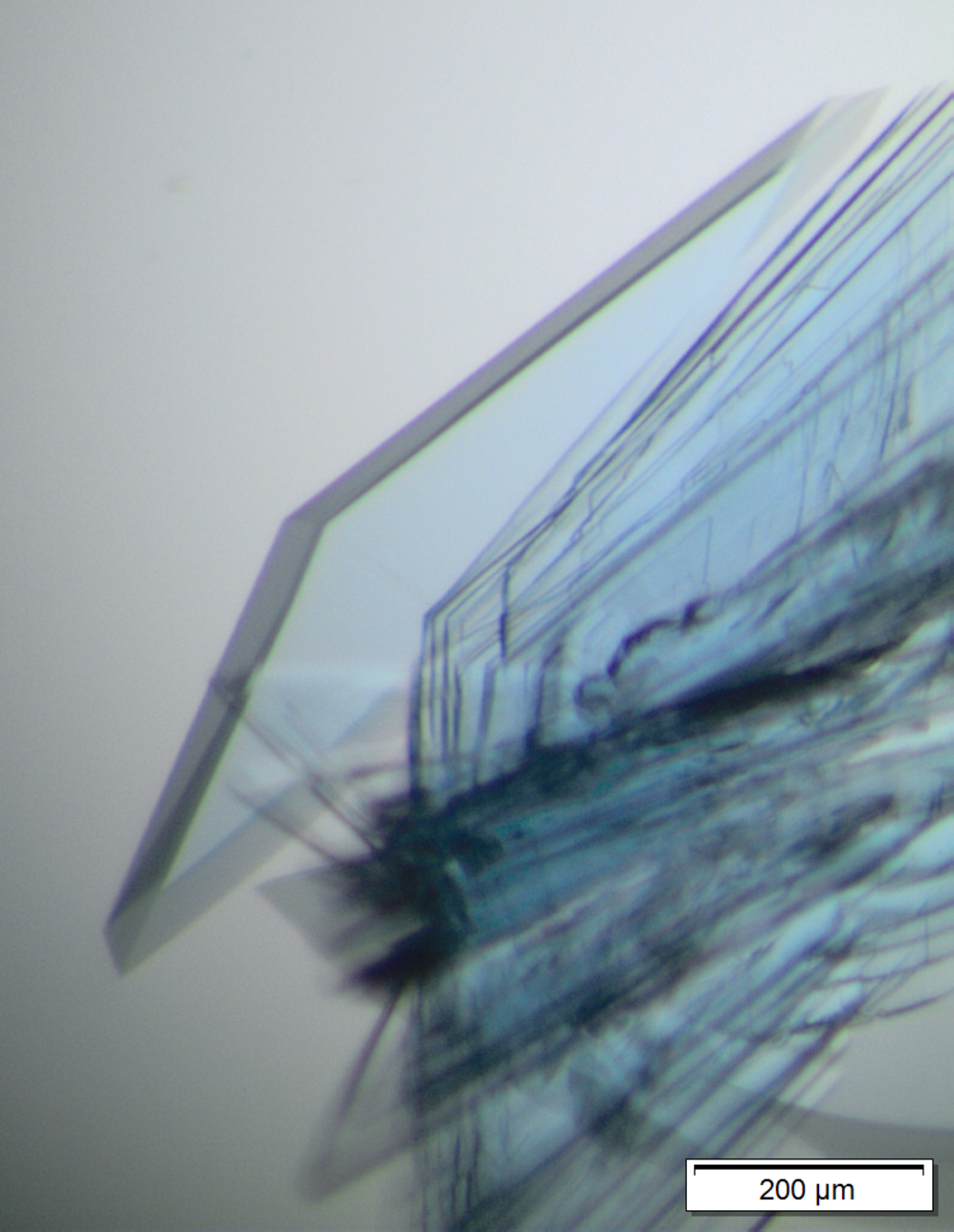

Supplement: S2 Fig — The large single crystal was one of the crystals used for data collection (approximate dimensions 700 x 200 x 30 μm). (TIF) [file pone.0206589.s002.tif]

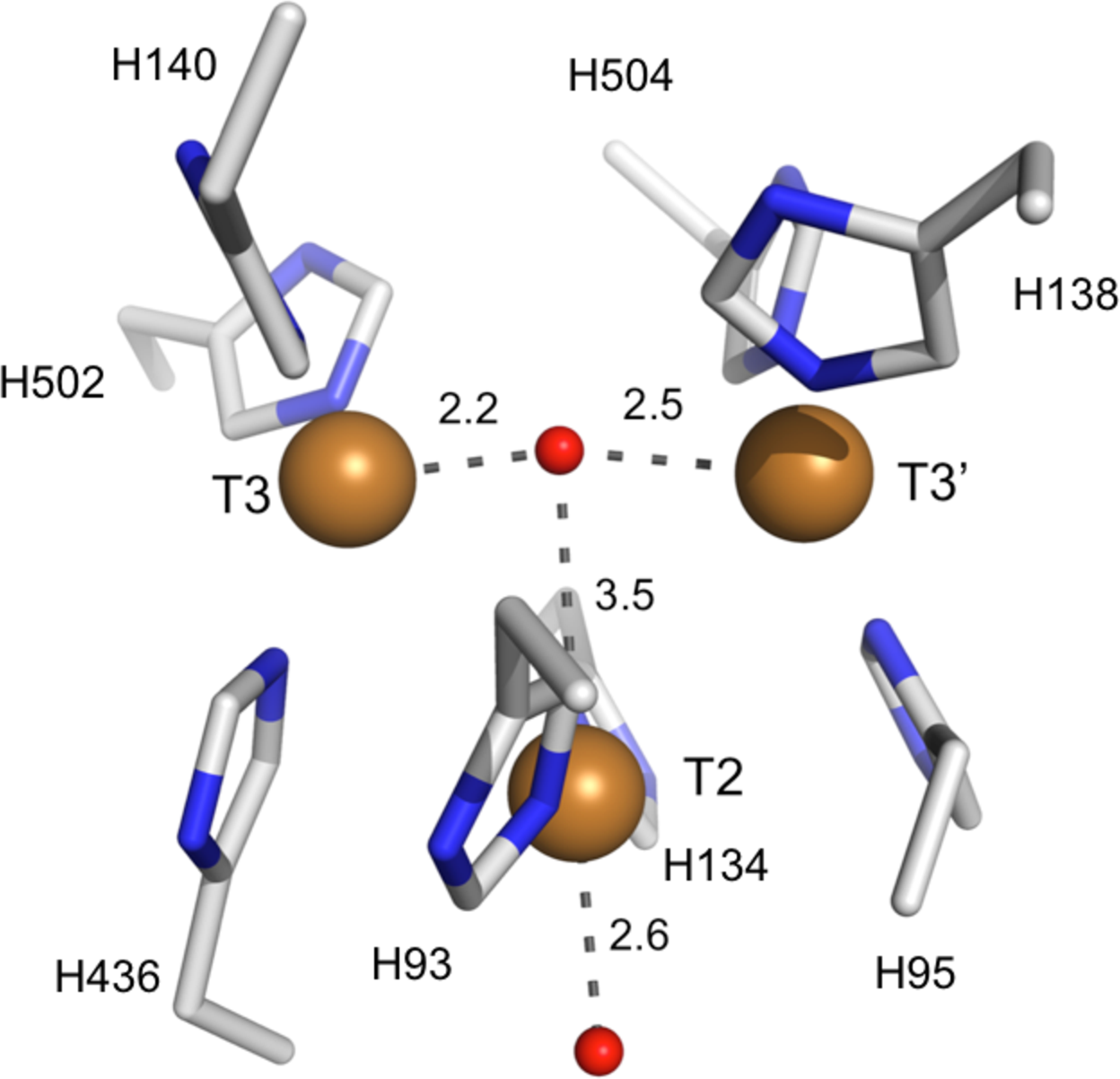

Supplement: S3 Fig — Classical resting state geometry with oxidized Cu ions, OH- bridging the T3-coppers and water/hydroxide coordinated to T2. The Cu-Cu-distance is 4.6 Å. (TIF) [file pone.0206589.s003.tif]

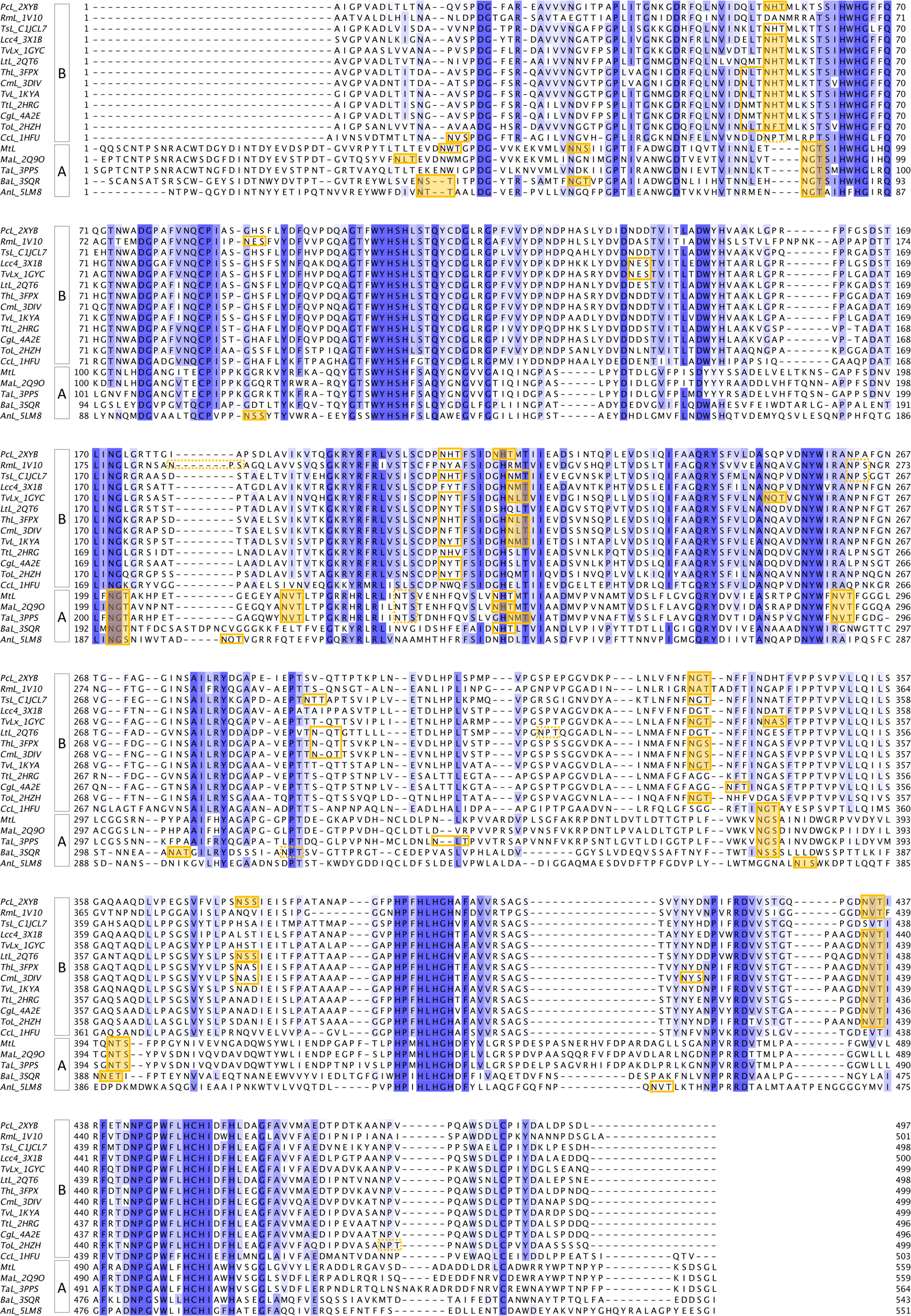

Supplement: S4 Fig — Mapping of N-glycosylation sites in selected asco- (A) and basi- (B) laccases. Structure-based alignment where putative N-glycosylation sites with the consensus motif Asn-X-Ser/Thr are highlighted with orange boxes. For sites where attached glycans are observed in the crystal structure, the box is filled. Sites that are unlikely to be glycosylated, as they are either buried inside the protein structure or contain a Pro residue at the X1 site (Asn-Pro-Thr/Ser), are indicated with boxes with a dotted line. The alignment was made with Promals3D [42, 43] and is based on sequences and corresponding crystal structures of laccases from the basi-laccases Pycnoporus cinnabarinus (PcL, 2XYB), Rigidoporus microporus (RmL, 1V10), Trametes sanguinea (TsL; no 3D structure), Lentinus sp. (Lcc4, 3X1B), Trametes versicolor (TvLx, 1GYC), Lentinus tigrinus (LtL, 2QT6), Trametes hirsuta (ThL, 3FPX), Cerrena maxima (CmL, 3DIV), Trametes versicolor (TvL, 1KYA), Trametes trogii (TtL, 2HRG), Coriolopsis gallica (CgL, 4A2E), Trametes ochracea (ToL, 2HZH), Coprinus cinereus (CcL, 1HFU) and asco-laccases from Myceliophthora thermophila (MtL, 6F5K), Melanocarpus albomyces (MaL, 2Q9O), Thielavia arenaria (TaL, 3PPS), Botrytis aclada (BaL, 3SQR) and Aspergillus niger (AnL, 5LM8). No glycans were modelled in the RmL structure (1V10), but two sites were identified by MS-analysis [63]. No crystal structure is available for TsL (UNIPROT C1JCL7), but this sequence is included as this is the laccase used in the (de)-glycosylation studies by Vite-Vallejo and coworkers [20]. (TIF) [file pone.0206589.s004.tif]

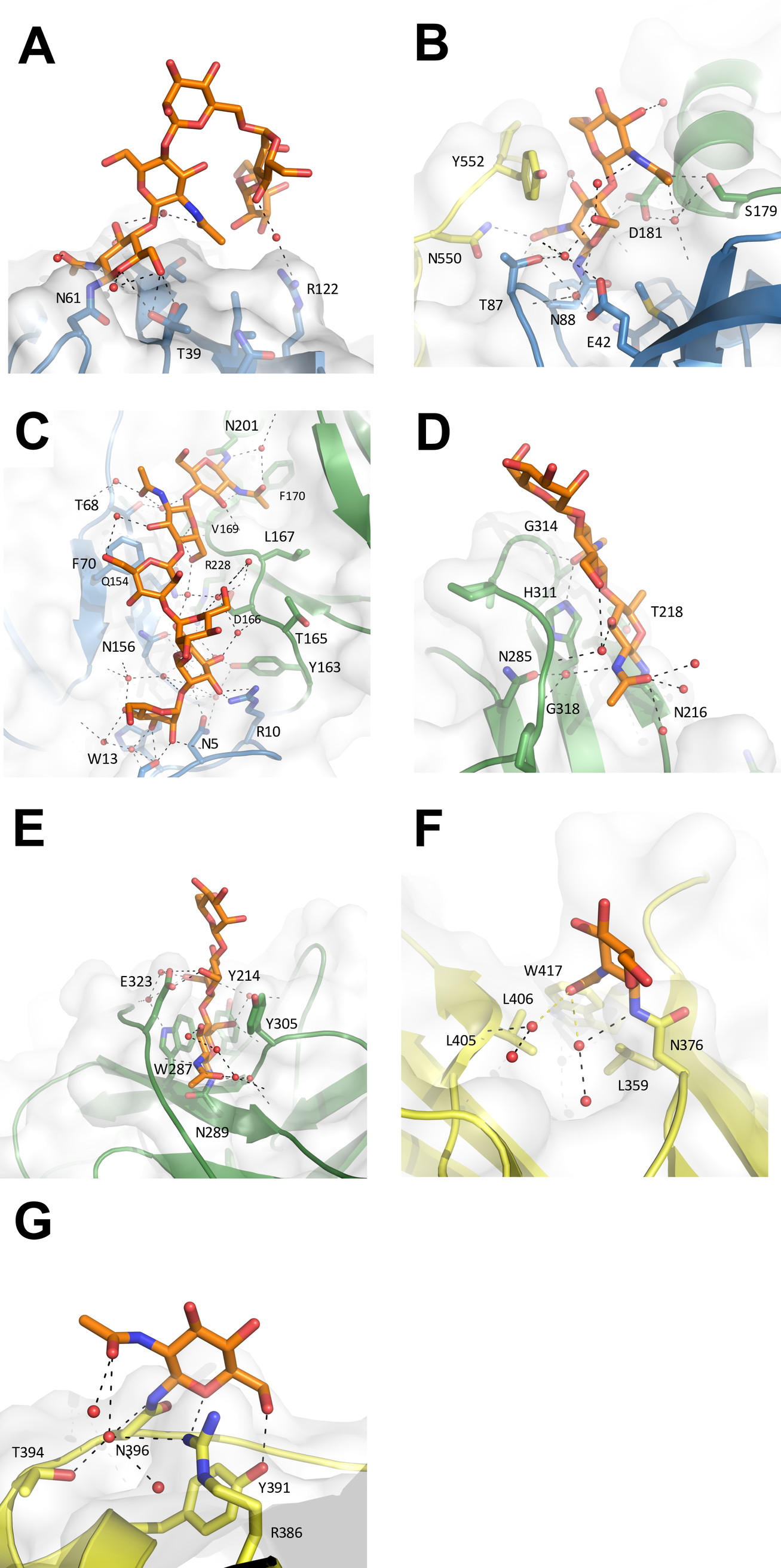

Supplement: S5 Fig — N-glycosylation sites A) Asn61, B) Asn88, C) Asn201, D) Asn216, E) Asn289, F) Asn376 and G) Asn396. (TIF) [file pone.0206589.s005.tif]
