# Supplementary material for: A comparative structural analysis of the surface properties of asco-laccases
Source: PLoS One. 2018 Nov 5;13(11):e0206589. doi: 10.1371/journal.pone.0206589 (PMC6218047; doi:10.1371/journal.pone.0206589)
Supplement: S1 Table — (PDF) [file pone.0206589.s006.pdf]

S1 Table. Asco-laccases with known structure

	Organism	Order	PDB entry	Resolution (Å)	Reference
*Mt*L	*Myceliophthora thermophila*	Sordariomycetes	6F5K	1.6	This study
*Ma*L	*Melanocarpus albomyces*	Sordariomycetes	2Q9O	1.3	(1)
*Ta*L	*Thielavia arenaria*	Sordariomycetes	3PPS	2.5	(2)
*Ba*L	*Botrytis aclada*	Leotiomycetes	3SQR	1.7	(3)
*An*L	*Aspergillus niger*	Eurotiomycetes	5LM8	1.7	(4)

1. Hakulinen N, Andberg M, Kallio J, Koivula A, Kruus K, Rouvinen J. A near atomic resolution structure of a Melanocarpus albomyces laccase. J Struct Biol. 2008;162(1):29-39.
2. Kallio JP, Gasparetti C, Andberg M, Boer H, Koivula A, Kruus K, et al. Crystal structure of an ascomycete fungal laccase from Thielavia arenaria--common structural features of asco-laccases. FEBS J. 2011;278(13):2283-95.
3. Osipov E, Polyakov K, Kittl R, Shleev S, Dorovatovsky P, Tikhonova T, et al. Effect of the L499M mutation of the ascomycetous Botrytis aclada laccase on redox potential and catalytic properties. Acta Crystallogr D Biol Crystallogr. 2014;70(Pt 11):2913-23.
4. Ferraroni M, Westphal AH, Borsari M, Tamayo-Ramos JA, Briganti F, Graaff LHd, et al. Structure and function of Aspergillus niger laccase McoG. Biocatalysis. 2017;3(1):1-21.
