# Supplementary material for: A comparative structural analysis of the surface properties of asco-laccases
Source: PLoS One. 2018 Nov 5;13(11):e0206589. doi: 10.1371/journal.pone.0206589 (PMC6218047; doi:10.1371/journal.pone.0206589)
Supplement: S2 Table — (PDF) [file pone.0206589.s007.pdf]

S2 Table. Pairwise sequence identity (%) between asco-laccases.

Name	UNIPROT	# residues in mature enzyme	*Mt*L	*Ma*L	*Ta*L	*Ba*L
*Mt*L	G2QG31*	559				
*Ma*L	Q70KY3	559	75.3			
*Ta*L	F6N9E7	564	70.6	75.7		
*Ba*L	H8ZRU2	543	38.7	38.1	38.6	
*An*L	A2QS62	577	34.2	35.3	33.0	41.8

*The *Mt*L sequence corresponds to G2QG31 with six modifications: R62N followed by a four residue insert (SIIG) and H545R (*Mt*L numbering).

Overall sequence identity is 23 % (126/559); 66 % within the *Mt*L, *Ma*L and *Ta*L subgroup.
