# Supplementary material for: A comparative structural analysis of the surface properties of asco-laccases
Source: PLoS One. 2018 Nov 5;13(11):e0206589. doi: 10.1371/journal.pone.0206589 (PMC6218047; doi:10.1371/journal.pone.0206589)
Supplement: S3 Table — (PDF) [file pone.0206589.s008.pdf]

S3 Table. Structural alignment of *Mt*L with other asco-laccases.

Name	Pairs of C_α atoms aligned	r.m.s.d. (Å)	Cycle *
*Ma*L	521	0.60	1
	472	0.37	5
*Ta*L	522	0.78	1
	455	0.41	5
*Ba*L	491	3.69	1
	393	0.73	5
AnL	486	3.81	1
	359	0.82	5

*Cycle in *align* algorithm in PYMOL
